# Well-differentiated liver cancers reveal the potential link between ACE2 dysfunction and metabolic breakdown

**DOI:** 10.1038/s41598-021-03710-0

**Published:** 2022-02-03

**Authors:** Lise Desquilles, Luis Cano, Gevorg Ghukasyan, Nicolas Mouchet, Clémence Landreau, Anne Corlu, Bruno Clément, Bruno Turlin, Romain Désert, Orlando Musso

**Affiliations:** 1 INSERM, INRAE, University of Rennes, Nutrition Metabolisms and Cancer, Rennes, France; 2 University of Rennes, CNRS, INSERM, UMS Biosit, Core Facility H2P2, 35000 Rennes, France

**Keywords:** Bioinformatics, Epigenetics analysis, Gene expression analysis, Genomic analysis, Imaging, Immunological techniques, Microscopy, Sequencing

## Abstract

Angiotensin-converting enzyme 2 (*ACE2*) is the receptor of the Severe Acute Respiratory Syndrome Coronavirus 2 (SARS-CoV-2) causing Coronavirus disease 2019 (COVID-19). Transmembrane serine protease 2 (*TMPRSS2*) is a coreceptor. Abnormal hepatic function in COVID-19 suggests specific or bystander liver disease. Because liver cancer cells express the ACE2 viral receptor, they are widely used as models of SARS-CoV-2 infection in vitro*.* Therefore, the purpose of this study was to analyze ACE2 and TMPRSS2 expression and localization in human liver cancers and in non-tumor livers. We studied *ACE2* and *TMPRSS2* in transcriptomic datasets totaling 1503 liver cancers, followed by high-resolution confocal multiplex immunohistochemistry and quantitative image analysis of a 41-HCC tissue microarray. In cancers, we detected ACE2 and TMPRSS2 at the biliary pole of tumor hepatocytes. In whole mount sections of five normal liver samples, we identified ACE2 in hepatocyte’s bile canaliculi, biliary epithelium, sinusoidal and capillary endothelial cells. Tumors carrying mutated β-catenin showed *ACE2* DNA hypomethylation and higher mRNA and protein expression, consistently with predicted β-catenin response sites in the *ACE2* promoter. Finally, *ACE2* and *TMPRSS2* co-expression networks highlighted hepatocyte-specific functions, oxidative stress and inflammation, suggesting a link between inflammation, ACE2 dysfunction and metabolic breakdown.

## Introduction

Severe Acute Respiratory Syndrome Coronavirus 2 (SARS-CoV-2) causes the Coronavirus disease 2019 (COVID-19)^1–3^. SARS-CoV-2 is an enveloped RNA betacoronavirus phylogenetically similar to SARS-CoV and the Middle East Respiratory Syndrome Coronavirus (MERS-CoV). Although ~ 80% of SARS-CoV-2-infected individuals develop asymptomatic self-limited upper airway forms of COVID-19, approximately 15% need hospitalization and 5% develop severe disease with acute respiratory distress syndrome, immune dysregulation and a “cytokine storm” with disseminated intravascular coagulation^1,3,4^. Approximately 50% of hospitalized patients present comorbidities: hypertension, type-2 diabetes and coronary heart disease^1–3,5,6^.

The baseline prevalence of chronic liver disease in COVID-19 was about three percent in two meta-analyses assembling 3301 patients^7,8^, but the mean prevalence of COVID-19 related liver dysfunction was about 20% (range 14–53%)^1,6–9^. Liver dysfunction correlates with the extent of pulmonary lesions^10^. Patients with hepatocyte-type dysfunction at admission are at higher risk of progressing to severe COVID-19^11^. After admission, antiviral drugs such as lopinavir and ritonavir are risk factors for liver damage^11^. Autopsies suggested that SARS-CoV-2 infects and replicates within hepatocytes in severe COVID-19 cases^12–14^ and SARS-CoV-2 RNA has been detected in the respiratory epithelium, kidney, liver, heart, brain, blood and stools in severe COVID-19 cases^15,16^. In addition, crown-like viral particles with complete envelope and typical spikes have been observed in lung, kidney^16,17^ and in hepatocytes^14^ suggesting multiorgan targeting of COVID-19.

Cell entry of SARS-CoV and SARS-CoV-2 depends on the binding of the viral spike (S) protein to the Angiotensin-converting enzyme 2 (ACE2) and on the serine protease TMPRSS2 for S protein fusion^18,19^. The rate of SARS-CoV-2 infection correlates with cell surface ACE2 expression^18,19^. Importantly, Huh-7^18,20^ human hepatocellular carcinoma (HCC) and HepG2 hepatoblastoma^20^ cells are widely used models of SARS-CoV-2 infection because they express cell surface ACE2 and can be infected at high titers.

As liver cancer cells are in vitro models for the study of SARS-CoV-2 entry and cytopathic effects, we extensively studied mRNA, protein expression and localization of the viral receptors ACE2 and TMPRSS2 in human HCCs. This characterization will be useful to design in vitro studies on virus entry, replication and metabolic alterations in liver cells.

## Results

### Higher expression of *ACE2* and T*MPRSS2* mRNAs in HCCs with mutated β-catenin

We first searched for relationships of *ACE2* and *TMPRSS2* mRNA expression levels with HCC aggressiveness. To this end, overall and disease-free survival analyses were carried out in the TCGA dataset (*n* = 370). After applying the exclusion criteria described in Supplementary Fig. 1a, the survival dataset consisted of 256 patients. In consistency with previous reports^21–23^, higher *ACE2* and *TMPRSS2* mRNA expression were associated with better overall and disease-free survival (Supplementary Fig. 1b and 2a). *ACE2* and *TMPRSS2* mRNAs were also detected in 47 non-tumor livers in the TCGA dataset. Whereas *ACE2* mRNA expression was 1.7-fold higher in non-tumors livers than in HCCs, *TMPRSS2* expression did not differ between both groups (Supplementary Fig. 2b).

**Figure 1:**
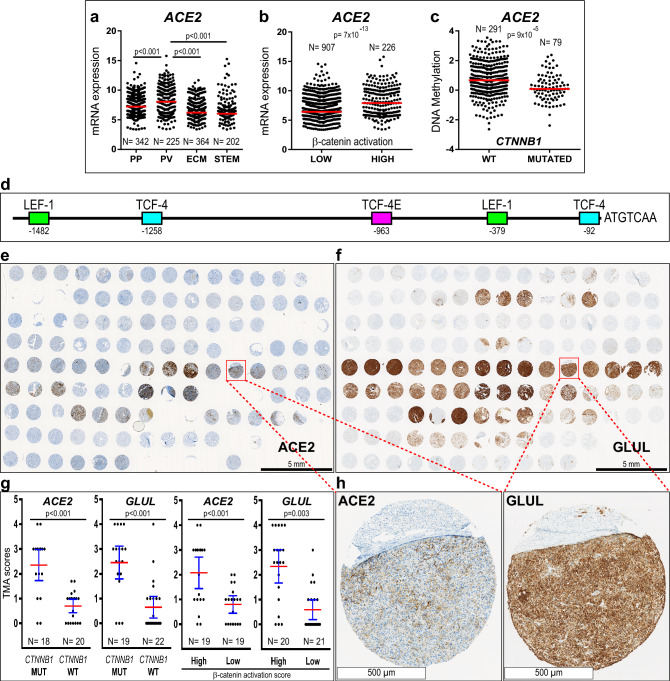
High ACE2 levels in well-differentiated, non-proliferative HCCs, with mutated β-catenin (*CTNNB1*) and hypomethylated DNA. (**a**) and (**b**) *ACE2* mRNA expression in the Désert’s meta-dataset of 1133 HCCs^26^. (**a**) HCC subclasses: PP, periportal-type; PV, perivenous-type; ECM, extracellular-matrix-type and STEM, stem-cell-type HCCs. (**b**) *ACE2* mRNA expression according to β-catenin activation levels, assessed as described^26^. (**c**) *ACE2* DNA is hypomethylated in HCCs carrying mutated *CTNNB1* (TCGA dataset). (**d**) TCF/LEF-1 binding sites, responding to β-catenin transcriptional activation in the proximal *ACE2* DNA sequence upstream the transcription start site, according to the PROMO program (TRANSFAC database). (**e**) and (**f**) Immunohistochemical detection (*brown signal*) of ACE2 (**e**) and GLUL (glutamine synthetase, (**f**) in an HCC tissue microarray (*TMA*). Slides were slightly counterstained with hematoxylin (*blue*). Three 1-mm in diameter spots were punched from each formalin-fixed paraffin-embedded routine liver tissue block (*n* = 41 HCCs; 2 normal liver controls). Digital slides were acquired with a 20X objective. (**g**) ACE2 and GLUL immunohistochemical signal scoring in HCCs, according to the tumor’s *CTNNB1* mutational status, (*MUT*) *versus* wild-type (*WT*); and β-catenin activation scores, (*High*) *versus* (*Low*), using the median value as a cut-off (*High,* β-catenin activation score > 4). TMA scores are shown as mean (red bar) ± 95% confidence intervals (blue bars). Each dot corresponds to the average out of triplicate tissue cores from each HCC. Statistical differences between means calculated with Mann–Whitney *U* test. Tumor numbers for each group are indicated. Only were scored those HCCs for which at least two spots were exploitable. TMA^33^ and β-catenin activation^26^ scoring were performed as we previously described. Average GLUL and ACE2 scores, *CTNNB1* mutational status and β-catenin activation scores for each HCC are provided in Supplementary Table 5. **(h)** Higher magnification from the indicated spots in (**e**) and (**f**)*.*

In HCCs, the expression of genes associated with relatively good prognosis is frequently preserved in well-differentiated tumors because they maintain the phenotype of adult hepatocytes^24,25^. To verify if this applied to *ACE2* and *TMPRSS2,* we measured their expression levels in HCCs classified according to their likeness to normal hepatocytes. Thus classified, HCCs are divided into two major classes, namely “low-proliferation” and “high-proliferation”^25^. Four HCC subclasses result from the interaction of, on one hand, the metabolic phenotype and, on the other hand, the proliferation/differentiation ratios of tumor cells^24,26^. Hence, in the “low-proliferation” class of well-to-moderately differentiated HCCs, the periportal-type (PP) and perivenous-type (PV) subclasses refer to the preservation of the metabolic zonation functions of normal hepatocytes^24,26^. At the opposite end of the spectrum, in the “high-proliferation” class of moderately-to-poorly differentiated HCCs, the extracellular-matrix-type (ECM) and STEM-type subclasses, refer to tumors developing an important stromal and vascular support and expressing cancer stem cell markers^24,26^. In this context, we found higher *ACE2* mRNA expression in perivenous-type HCCs than in the other subclasses (Fig. 1a; Supplementary Fig. 2c) in both the Désert’s cDNA microarray meta-dataset (1133 HCCs)^26^ (Fig. 1a) and in the TCGA dataset (370 HCCs) (Supplementary Fig. 2c). Of note, *ACE2* mRNA expression was higher in HCCs expressing previously validated β-catenin pathway activation signatures^26,27^ (Fig. 1b) and in HCCs carrying sequenced β-catenin (*CTNNB1*) activating mutations (Supplementary Fig. 2c). In turn, *TMPRSS2* was expressed at higher levels in periportal-type HCCs (Supplementary Fig. 2c, d), which we previously demonstrated to carry wild-type *CTNNB1* and to belong to the “low-proliferation” class of well-to-moderately differentiated HCCs that preserve the periportal metabolic functions of normal hepatocytes^26^. The MERS-CoV receptor, *DPP4,* was also related to β-catenin pathway activation (Supplementary Fig. 2e–g). *ACE2* DNA was hypomethylated in tumors carrying *CTNNB1* mutations (Fig. 1c), which is consistent with transcriptional activation the *ACE2* gene*.* By contrast, *TMPRSS2* was hyper-methylated in HCCs carrying *CTNNB1* mutations (Supplementary Fig. 3a; Supplementary Table 1), which is consistent with transcriptional repression*.* Neither *ACE2* nor *DPP*4 mRNA expression were related *TERT* and *TP53* gene mutations, which are the two most frequent mutations in HCCs (Supplementary Fig. 3c, d). In turn, *TMPRSS2* mRNA levels were higher in HCCs carrying wild-type *TP53* (Supplementary Fig. 3d), which is consistent with the above-described higher expression of *TMPRSS2* in the well-differentiated, non-proliferative class of HCCs, where *TP53* gene mutations are rare^26,28^. Indeed, *TP53* mutations are most frequent in poorly-differentiated HCCs (S2^29^; G3^30^), showing an aggressive, cancer stem cell phenotype^26,31^.

In line with the higher *ACE2* and *DPP4* mRNA expression in HCCs showing increased β-catenin pathway activation and *CTNNB1* activating mutations, in silico analysis of 5,000 base pairs upstream of the transcription start sites of *ACE2* and *DPP4* DNAs revealed putative T-cell factor 4/LEF-1 consensus transcription factor binding sites, respectively (Fig. 1d; Supplementary Fig. 4a, b).

### ACE2 is predominantly immunodetected in perivenous-type HCCs carrying *CTNNB1* mutations and preserving hepatocyte polarization

In consistency with the above findings, scoring of immunohistochemical signal for ACE2 and for the β-catenin target gene GLUL (glutamine synthetase) in a tissue microarray of 41 HCCs (Fig. 1e, f; Supplementary Table 5) revealed that the expression of ACE2 and GLUL were correlated (Spearman’s R = 0.62; p < 0.001; n = 38). Sanger sequencing of the mutational hotspot in the 3rd exon of *CTNNB1* revealed that HCCs carrying *CTNNB1* activating mutations expressed 5.1- and 3.3-fold higher levels of GLUL and ACE2 proteins than those carrying wild-type *CTNNB1* (Fig. 1e–h; Supplementary Table 5). To confirm whether tumors expressing the highest levels of ACE2 protein corresponded to perivenous-type HCCs, we applied our previously described β-catenin pathway activation score^26^. It results from an equation based on mRNA expression data of five genes as follows: β-catenin activation score = [(*GLUL* ×* LGR5* ×*ODAM*)* ÷ *(*VNN1* × *HAL*)]^26^. Thus, we measured mRNA expression for these five genes by real-time PCR on mirror frozen samples from the same HCCs as those included in the TMA, applying our previously described method^26,32,33^. GLUL and ACE2 protein expression were correlated with β-catenin activation scores (GLUL, R = 0.69, *p* < 0.001, *n* = 41; ACE2, R = 0.51, *p* = 0.001, *n* = 38). β-catenin activation scores ranged from  − 2 to 20, median = 4 (Supplementary Table 5). As previously described^26^, high scores (above the median), define perivenous-type HCCs. On average, GLUL and ACE2 proteins were respectively 4.0- and 2.6-fold higher in HCCs with high β-catenin activation scores (Fig. 1g). These results indicate that HCCs preserving a perivenous-type metabolic phenotype express higher level of ACE2 protein^26^.

**Figure 2:**
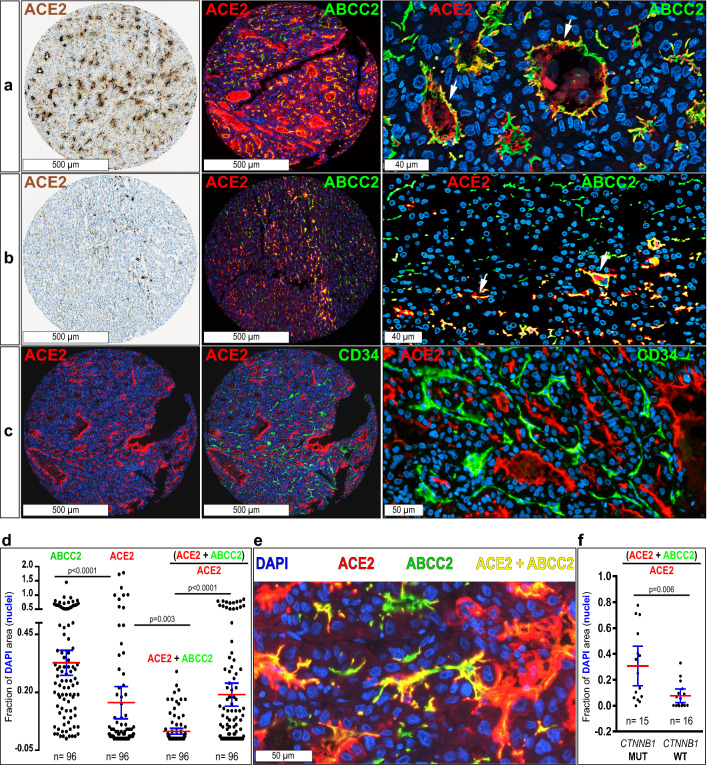
ACE2 is detected in the biliary pole of moderately-to-well-differentiated HCCs with trabecular or pseudo-glandular patterns. Combined immunoperoxidase and immunofluorescence analysis of a tissue microarray containing 41 HCCs and two normal liver controls, spotted in triplicates. Representative images are shown. Immunoperoxidase signal (*brown*) is counterstained with hematoxylin (*blue*)*.* ACE2 appears in *red* by immunofluorescence, other markers in *green* and nuclei in *blue* (DAPI staining). (**a**), (**b**) ACE2 colocalizes with the apical hepatocyte marker ABCC2 (a.k.a. MRP2) (*arrows*). (**c**) Tumor capillary vessels are marked with CD34. Confocal digital images were acquired with a 40X objective. Images are Z-stacks of four 500 nm focusing steps. (**d**) Quantification of ACE2, ABCC2, dual (ABCC2 + ACE2) staining and the ratio of (ACE2 + ABCC2) × ACE2^–1^, representing the fraction of ACE2 protein expression detected as dual (ACE2 + ABCC2) staining, *i.e.,* the fraction of overlap of ACE2 with ABCC2. Values for individual TMA spots from 32 HCCs are expressed as the fraction of DAPI staining of nuclei, to correct for natural variations in cell density in each spot. Means ± 95% confidence intervals are shown for each condition. The statistical significance between groups was calculated with Kruskal–Wallis test (*p* < 0.0001), followed by the post-hoc Dunn’s test as indicated. (**e**) Sample scan from a TMA spot showing examples for the different signals quantified in (**d**). (**f**) The fraction of overlapping (ACE2 + ABCC2) over total ACE2 signal (*i.e.,* HCC cells expressing ACE2 at the biliary pole), is higher in HCCs carrying *CTNNB1* mutations. Each data point represents the mean of two to three cores for each tumor. Statistical significance calculated by Mann–Whitney U test.

HCCs with mutated *CTNNB1* belong to the class of well-to-moderately differentiated tumors that preserve adult hepatocyte features^25^. Thus, they are frequently cholestatic and contain pseudo-glandular structures with bile plugs, where hepatocytes are polarized and preserve the basal and apical poles^31^. Co-immunolabeling for ACE2 and ABCC2 (a.k.a. MRP2, a marker of the biliary pole in hepatocytes) in HCCs, detected ACE2 at the biliary pole and within the lumen of pseudo-glandular tumor structures and in slit-like bile canaliculi (Fig. 2a,b). By contrast, ACE2 was not detected at the basal hepatocyte pole, which was highlighted by the capillary endothelial cell marker CD34 (Fig. 2c). To quantify ACE2 protein expression at the biliary pole of HCC cells, we measured the fluorescence area for separate ACE2 and ABCC2 signals and for dual (ACE2 + ABCC2) staining in 96 TMA spots corresponding to 32 HCCs. To correct for differences in cell densities among HCCs, we plotted specific signal areas as the fraction of the DAPI area (*i.e.,* nuclear staining) (Fig. 2d,e). Mean ± SD ABCC2/DAPI or ACE2/DAPI ratios were: ABCC2/DAPI, 33 ± 27%; ACE2/DAPI, 16 ± 34%; (ACE2 + ABCC2)/DAPI, 3 ± 6%. Of note, 19 ± 25% of all ACE2 expression (i.e., (ACE2 + ABCC2)/ACE2) was located at the biliary poles of HCC cells (Fig. 2d,e).

In tumors without *CTNNB1* mutations, only 8 ± 9% of all ACE2 was localized at the biliary poles. By contrast, in tumors with mutated *CTNNB1,* 31 ± 27% of all ACE2 was detected at the biliary poles (Fig. 2f) and the mean dual (ACE2 + ABCC2)/DAPI signal was 35 folds higher in HCCs carrying mutated than in tumors carrying wild-type *CTNNB1* (mean ± SD: wild-type, 0.0018 ± 0.004; mutated, 0.063 ± 0.07; *p* = 0.002), thus confirming that HCCs carrying mutated *CTNNB1* are enriched in ACE2 expression and indicating that ACE2 is particularly associated with the biliary pole in tumor cells preserving polarized hepatocyte-like features. Adding robustness to these data, dual (ACE2 + ABCC2) expression was correlated with β-catenin activation scores (Spearman’s R = 0.58, *n* = 31; *p* = 0.0005).

TMPRSS2 was also detected at the apical pole of some of the pseudo-glandular formations or within slit-like trabecular structures and in cell–cell borders (Supplementary Fig. 5). In contrast with ACE2, the signal for TMPRSS2 was mild in HCCs, suggesting low protein expression levels, which were unrelated to *CTNNB1* mutations. Neither ACE2 nor TMPRSS2 (not shown) were detected in myofibroblasts as suggested by dual ACE2 and ACTA2 staining (Supplementary Fig. 5).

**Figure 3:**
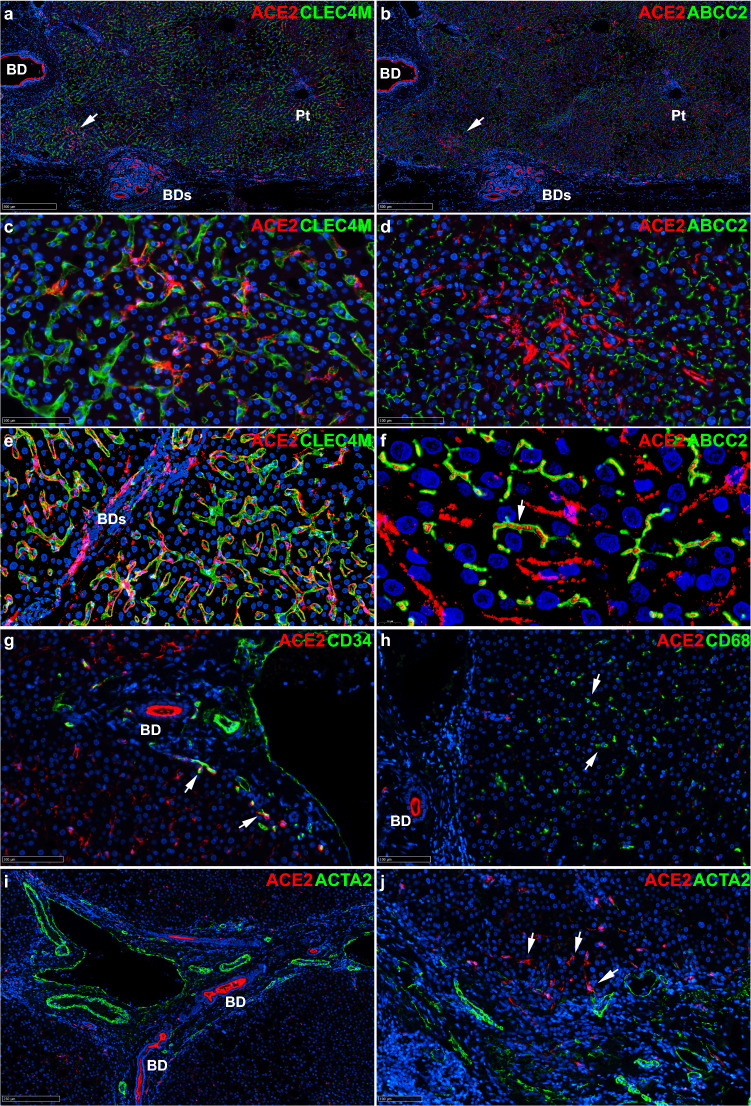
ACE2 is detected in sinusoidal endothelial cells, bile ducts and capillaries of the periportal plexus in non-tumor livers. Co-immunostaining for ACE2 and the indicated markers in three non-tumor livers from patients undergoing resection of colon cancer metastasis. Representative images are shown. ACE2 appears in *red* by immunofluorescence, other markers in *green* and nuclei in *blue* (DAPI staining). (**a**), (**b**) Low power views (X5 magnification) show ACE2 in a bile duct (*BD*) and in a zone with mild inflammatory infiltration (*arrow*) and dystrophic bile ducts (*BDs*). The sinusoidal endothelial cell marker CLEC4M (a.k.a. DC-SIGNR) and the bile canaliculi marker ABCC2 (a.k.a. MRP2) show lobular staining at this magnification. *Pt,* portal tract. (**c**), (**d**) At X30 magnification, ACE is detected in sinusoidal endothelial cells, which can be identified by comparing CLEC4M (**c**) and ABCC2 (**d**) staining. Digital images were acquired with a microscope scanner and a X40 objective and exported at the indicated magnifications (Nanozoomer, Hamamatsu Photonics). (**e**), (**f**) Confocal scans of whole tissue sections as Z-stacks of 4 X 500 nm focusing steps at X40 (**e**) and X143 (**f**). ACE2 is detected in CLEC4M-positive sinusoidal endothelial cells (**e**) and within ABCC2-positive bile canaliculi (**f**). ACE2-positive bile ducts (*BDs*) are seen in (**e**). (**g**) At 28X magnification, ACE2 is sparsely detected in CD34-positive endothelial cells of the periportal vascular plexus (*arrows*)*.* (**h**) At 24X magnification, no evidence of ACE2 detection in CD68-positive Kupffer cells. (**i**), (**j**) No evidence of ACE2 detection in vascular smooth muscle (**i**, 10X magnification) or in ACTA2 (a.k.a alpha smooth muscle actin)-positive cells within portal tract stroma (**j**, 20X magnification). ACE2-positive cells within the portal tract are ACTA2 negative (*arrows*)*.*

In five non-tumor samples from patients undergoing resection of colon cancer metastases and with minimal inflammatory changes in the liver, ACE2 was co-detected with the sinusoidal endothelial cell marker CLEC4M (a.k.a. DC-SIGNR) (Fig. 3a,c,e) and the bile canaliculi marker ABCC2 (a.k.a. MRP2) (Fig. 3b,d,f). The presence of ACE2 within bile canaliculi is consistent with the facts that the extracellular part of ACE2 can be cleaved off the cell surface by ADAM17, thus shedding functional ACE2 fragments^34^; and that ACE2 is an abundant component of the normal human bile proteome^35^. Also, ACE2 was co-detected with CD34 in capillary vessels of the periportal vascular plexus (Fig. 3g). By contrast, ACE2 was not detected in CD68-positive sinusoidal cells (Kupffer cells, Fig. 3h) or ACTA2-positive vascular smooth muscle cells or myofibroblasts (Fig. 3i,j). As expected, the antibodies used detected high levels of ACE2 in the apical intestinal epithelium, Bowman’s capsule and convoluted tubes in the kidney; in turn, TMPRSS2 was detected in the apical compartment of the epithelial lining of prostatic glands (Supplementary Fig. 5).

In summary, dual labeling of HCC and of non-neoplastic liver with cell-type specific antibodies showed ACE2 in the epithelial lining of bile ducts and in bile canaliculi, *i.e.,* the biliary pole of normal and differentiated tumor hepatocytes. In non-neoplastic liver, CLEC4M-positive sinusoidal endothelial cells and CD34-positive capillary endothelial cells of the periportal vascular plexus also contain ACE2.

### *ACE2* and *TMPRSS2* co-expression networks highlight metabolic functions typical of normal hepatocytes

**Figure 4:**
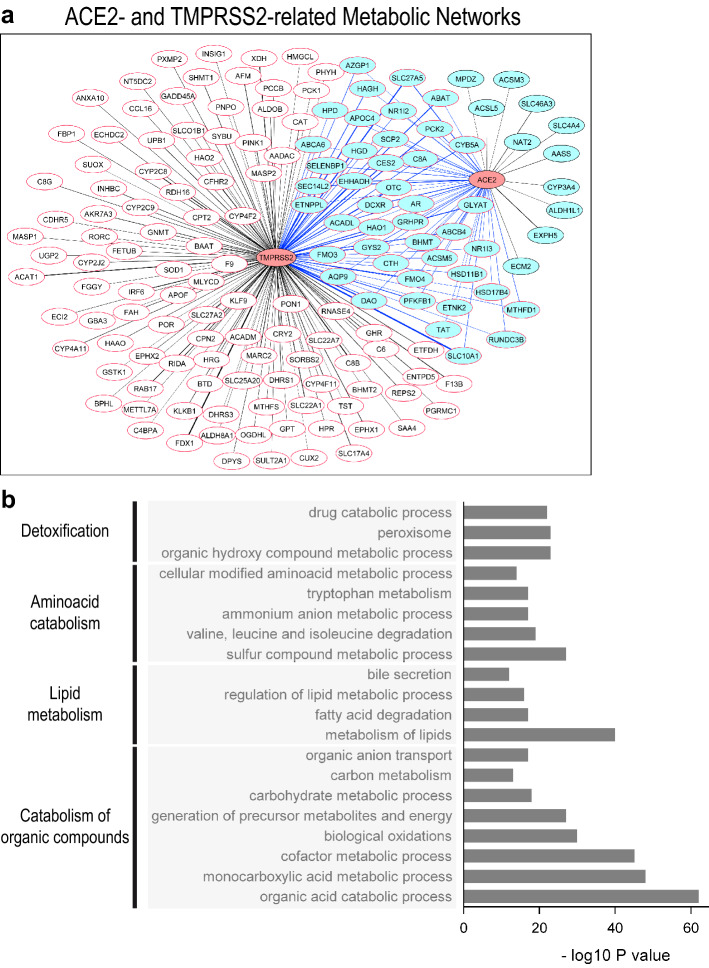
(**a**) Connections between the *ACE2* and the *TMPRSS2* expression networks in the Désert’s meta-dataset^26^, in non-proliferative-moderately-to-well-differentiated, periportal-type and perivenous-type HCCs^24^. The co-expression network was identified by Weighted Gene Correlation Network Analysis and the *WGCNA*^36^ R package, followed by visualization with Cytoscape^49^. Genes co-expressed with *ACE2, light-blue nodes with black borders and black links*; genes co-expressed with *TMPRSS2, white nodes with red borders and black links*; genes co-expressed with both *ACE2* and *TMPRSS2, light-blue nodes with red borders and blue links.* Link thickness is proportional to the correlation coefficient between a given node and *ACE2* or *TMPRSS2.* (**b**) Functional analysis and gene ontology enrichment of the gene co-expression network shown in *b* are visualized with Express Analysis from Metascape^50^. Gene ontology (GO) terms, families and *P* values are indicated.

By Weighted Gene Correlation Network Analysis^36^ comparing periportal-type (*n* = 342) and perivenous-type (*n* = 225) HCCs within the Désert’s dataset (*n* = 1133), we found that *ACE2* and *TMPRSS2* were related through interacting metabolic functions (Fig. 4a), including detoxification, aminoacid catabolism, lipid metabolism, fatty acid oxidation and catabolism of organic compounds, which is probably related to the carboxypeptidase functions of ACE2, involved in the degradation of inflammatory polypeptides^37,38^ (Fig. 4b). These findings are in line with the above-described expression of *ACE2* and *TMPRSS2* in well-to-moderately differentiated HCCs preserving the metabolic programs of adult hepatocytes. Next, we specifically investigated the *ACE2* co-expression network in HCCs and analyzed associated gene functions (Supplementary Tables 2, 3, 4). Not surprisingly, *ACE2* was associated with hepatocyte-specific functions such as aminoacid metabolism, xenobiotic detoxification, fatty acid uptake and oxidation. High *ACE2* expression was associated with low expression levels of oxidative stress markers and cytokine-mediated inflammatory signals (Supplementary Fig. 7a, b). These findings raise the hypothesis of a link between inflammation, ACE2 dysfunction and metabolic breakdown that may be relevant to the pathogenesis of COVID-19.

## Discussion

In this work, we show that ACE2 and TMPRSS2 mRNAs and proteins are detected at higher levels in well-to-moderately differentiated HCCs than in poorly differentiated tumors. In particular, ACE2 is detected at the biliary pole in tumors preserving hepatocyte polarization. By contrast, in non-tumor livers, ACE2 is detected not only in the epithelial lining of bile ducts and within hepatocyte’s bile canaliculi, but also in the CLEC4M-positive sinusoidal endothelium and the CD34-positive capillary vessels of the periportal plexus. TMPRSS2, in turn, is not detected in normal liver, but is detected at the biliary pole in well-to-moderately differentiated HCCs, which is consistent with the functional association of ACE2 and TMPRSS2 through periportal and perivenous metabolic networks. Of note, high-resolution 500-nm optical sections revealed ACE2 within hepatocyte’s bile canaliculi in histologically normal liver. This finding agrees with the high content of N-glycosylated, lectin-binding ACE2 in the normal human bile proteome^35^ and with the role of ACE2 in aminoacid uptake in the gut and in the regulation of the splanchnic blood flow^37^.

In HCCs carrying activating β-catenin (*CTNNB1*) mutations, *ACE2* DNA is hypomethylated, and *ACE2* mRNA expression upregulated. Conversely, *TMPRSS2* mRNA is lower in HCCs carrying mutated *CTNNB1* and its DNA hypermethylated. This finding agrees with the fact that only *ACE2* (and *DPP4,* but not *TMPRSS2*) contain TCF/LEF-1 binding sites that are predicted to respond to β-catenin transcriptional activation. Neither *ACE2,* nor *DPP4* or *TMPRSS2* are associated to *TERT* or *TP53* gene mutations. On the contrary, HCCs containing mutated *TP53* show relatively lower levels of *TMPRSS2* (Supplementary Fig. 3D). This association does not necessarily imply a direct regulation of *TMPRSS2* by *TP53,* but may be coincidental. In fact, well-differentiated, periportal-type HCCs express the highest levels of *TMPRSS2* and, as we previously showed, have the lowest prevalence of *TP53* mutations^26^. Nonetheless, the relationships of *ACE2* and *TMPRSS2* with histological, transcriptomic and genomic HCC subclasses converge to demonstrate that tumors preserving hepatocyte-like features, such as hepatocyte trabeculae with bile canaliculi and/or pseudo-glandular structures with an apical biliary pole, express higher levels of the SARS-CoV-2 receptor and co-receptor couple.

The above findings agree with further observations in this work: the *ACE2* co-expression network included liver-enriched genes involved in hepatocyte-specific functions, such as aminoacid metabolism (*GNMT*)*,* xenobiotic detoxification (*CYP3A4*)*,* fatty acid uptake, bile acid reconjugation (*SLC27A5*)*,* enterohepatic circulation of bile acids and cholesterol homeostasis (*SLC10A1*)*. ACE2* was also co-expressed with *SIRT1* and *CD36* and the fatty acid oxidation enzymes *ACADL, EHHADH, ACADM and ECI2,* and alternative glucose metabolism pathways (*CRYL1*)*,* as well as enzymes involved in gluconeogenesis (*PCK2*)*.* This network is in line with the role of ACE2 as a regulator of fatty acid uptake in hepatocytes through the angiotensin_1-7_-MAS1-SIRTUIN1-CD36 axis^39^. Indeed, *ACE2* knock-out mice show low levels of peripheral lipid uptake, but high hepatocyte uptake of triglycerides, resulting in steatosis, lipid peroxidation, oxidative stress and inflammation^39^. In turn, the induction of oxidative stress by *ACE2* deficiency may be related to its effects on glucose and lipid metabolism because the ACE2/Ang_1-7_/MAS axis increases glucose uptake, decreasing insulin resistance. Thus, *ACE2* knock-out mice exhibit high oxidative stress, whereas exposure to Ang_1-7_ reduces oxidative stress in liver cells through insulin signaling^40^. Taken together, this body of evidence matches the metabolic landscape of HCCs carrying activating *CTNNB1* mutations. These tumors oxidize fatty acids as a source of energy to feed the oxidative phosphorylation pathway^41^, but resist to oxidative stress^42^ and suppress inflammation^43^.

ACE2 expression was related to gene functions that smother inflammation. Indeed, ACE2 was related to low levels of the inflammation-related transcripts *BIRC3, MSN, MMP9, LTB, TNFRSF4, IL4R, CCL20* and *IL32,* among others (Supplementary Table 2). ACE2 exerts anti-inflammatory effects through carboxyterminal cleavage of vasoactive polypeptides, such as Apelin-13, which induces vasodilation, and des-arg9-bradykinin, a neutrophil chemoattractant^44^. In turn, deletion of *ACE2* leads to tissue injury and oxidative stress, through upregulation of the *AT1* receptor and inflammatory cytokines like *IL1β, IL6, IL17, RANTES, ICAM1, TNFα, TNFRSF1A* and downregulation of the anti-inflammatory cytokine *IL10*^45^. This picture is surprisingly similar to that seen in the spectrum going from immune dysregulation to macrophage activation syndrome in moderate-to-severe COVID-19^4,46^.

The anti-inflammatory role of ACE2 goes well with its up-regulation in response to liver injury, such as bile-duct ligation^47^ and with the evidence that ACE2 produced by hepatocytes limits fibrogenesis through degradation of AngII to Ang_1-7_^37^. In human liver cirrhosis, ACE2 protein is increased by > 90 folds in hepatocytes. In vitro*,* hypoxia increases ACE2 activity in hepatocytes, enhancing acetylcholine-induced vasodilatation^38^. As hypoxia activates Wnt/β-catenin signaling, it would be interesting to investigate whether ACE2 expression in perivenous hepatocytes depends on β-catenin^34,48^.

This body of matching data suggests that ACE2 may regulate energy metabolism, protecting against oxidative stress and inflammation. As both *TMPRSS2* and *ACE2* are functionally connected through metabolic pathways in the liver, it may be reasonable to hypothesize that ACE2 depletion resulting from SARS-CoV-2 infection^18^ may contribute to metabolic dysfunction and inflammation. Our results point to the relevance of further studies on the signaling pathways and pathological contexts leading to cell surface *ACE2* availability and to the possible impact of viral infection on the disruption of the *ACE2* metabolic network.

## Materials and methods

### Patients, samples and datasets

*Human hepatocellular carcinoma *(*HCC*)*:* immunohistochemistry was performed on a tissue microarray (TMA) built from formalin-fixed-paraffin-embedded (FFPE) routine tissue blocks of 41 HCC cases and two histologically normal liver controls, for which the exon 3 of β-catenin (*CTNNB1*) had been Sanger-sequenced, as we previously described^26^. In addition, three formalin-fixed, paraffin-embedded non-tumor liver tissue blocks from patients undergoing resection of liver metastases from extra-hepatic cancers were added to the immunohistochemistry analysis. To calculate β-catenin activation scores based on the mRNA expression of *GLUL, LGR5, ODAM, VNN1* and *HAL*, we retrieved real-time PCR mRNA expression data from our previously published work^26^ corresponding to frozen fragments of the 41 tumors arrayed in the TMA. Quality control matching of frozen and FFPE fragments has been previously described in an 80 HCC dataset including the 41-tumor subset^32,33^. Tissue samples and patient data were collected and anonymized after obtaining written informed consent from the patients. De-identification was performed in accordance with the Health Insurance Portability and Accountability Act (HIPAA) Privacy Rule. The study was approved by INSERM’s Institutional Advisory Board (approval number 19–630). Research involving human research participants has been performed in accordance with the Declaration of Helsinki. All experiments were performed in accordance with relevant guidelines and regulations. Two different immunohistochemical methods were used to detect primary antibodies: peroxidase-labeled secondary antibodies, revealed by a chromogenic substrate or multiplex immunofluorescence followed by analysis with a confocal slide scanner. The Désert’s microarray meta-dataset composed of 1133 human HCCs was previously described^26^. In this dataset, HCCs expressing a β-catenin-activated transcriptomic program were identified with a 5-gene signature composed of *GLUL, LGR5, ODAM, VNN1* and *HAL,* which predicts activating *CTNNB1* mutations with high sensitivity (0.86–0.91) and specificity (0.83–1.0)^26^. β-catenin pathway activation was confirmed with an independent 23-gene signature^27^. The publicly available TCGA dataset was composed of 370 HCCs and 47 matching non-tumor samples. RNA sequencing, mutation and methylation data were extracted as described^26^, using the *TCGAbiolinks* R package. Raw mRNA expression was median-normalized (*DESeq* R package). DNA methylation was quantile-normalized (*preprocessCore* R package); probes with B-value over 0.8 or under 0.2 were respectively considered as hypermethylated or hypomethylated. The R-package *Limma* was used to identify differential methylation.

See *Supplementary Information* for a detailed description of immunohistochemical methods and statistical analyses and Supplementary Table 6 for primary, secondary antibodies, fluorochromes used, epitope unmasking and incubation conditions.

## Supplementary Information

Supplementary Data.

Supplementary Figure 1.

Supplementary Figure 2.

Supplementary Figure 3.

Supplementary Figure 4.

Supplementary Figure 5.

Supplementary Figure 6.

Supplementary Figure 7.

Supplementary Table 1.

Supplementary Table 2.

Supplementary Table 3.

Supplementary Table 4.

Supplementary Table 5.

Supplementary Table 6.

## Data Availability

Datasets analyzed in this study are available at The Cancer Genome Atlas or at the Gene Expression Omnibus databases. Accession numbers and standard, publicly available R packages used are provided in the main text and supplementary files. The procedures applied for merging nine publicly available transcriptomic datasets into a meta-dataset totaling 1133 hepatocellular carcinomas have been previously published^26^. A filtered, normalized and batch-effect-corrected user-friendly version of this meta-dataset is available from the corresponding author upon reasonable request.

## Abbreviations

ACE2: Angiotensin-converting enzyme 2
Ang: Angiotensin
CLEC4M: C-Type Lectin Domain Family 4 Member M
DPP4: Dipeptidyl Peptidase 4
COVID-19: Coronavirus disease 2019
*CTNNB1*: β-Catenin
HCC: Hepatocellular carcinoma
LSECs, MERS-CoV: Middle East Respiratory Syndrome Coronavirus
SARS-CoV-2: Severe Acute Respiratory Syndrome Coronavirus 2
TCGA: The Cancer Genome Atlas
TMPRSS2: Transmembrane serine protease 2
