# Supplementary material for: Well-differentiated liver cancers reveal the potential link between ACE2 dysfunction and metabolic breakdown
Source: Sci Rep. 2022 Feb 3;12:1859. doi: 10.1038/s41598-021-03710-0 (PMC8814043; doi:10.1038/s41598-021-03710-0)
Supplement: Supplementary file 1 — Supplementary Data. [file 41598_2021_3710_MOESM1_ESM.pdf]

Well-differentiated liver cancers reveal the potential link between ACE2 dysfunction and metabolic breakdown.

Lise Desquilles,[1] Luis Cano,[1] Gevorg Ghukasyan,[2] Nicolas Mouchet,[2] Clémence Landreau,[1] Anne Corlu,[1] Bruno Clément,[1] Bruno Turlin,[1] Romain Désert,[1] Orlando Musso[1]*

[1]INSERM, INRAE, Univ Rennes, Nutrition Metabolisms and Cancer, Rennes, France.

[2]Univ Rennes, CNRS, INSERM, UMS Biosit, Core Facility H2P2, F-35000, France

---

SUPPLEMENTARY DATA

Supplementary Methods

Tissue microarrays

We have previously described the TMA scoring method in detail[1]. Briefly, TMA design and built were done with a MiniCore3 tissue arrayer equipped with a MiniCore control station and a TMADesigner2 tissue array design software (Aphelys, Plaisir, France). Triplicate, one millimeter in diameter cores were punched from each formalin-fixed-paraffin-embedded routine tissue block and inserted in a TMA receiver paraffin block. After paraffin sealing of tissue cores and block cooling, 5-µm microtome sections were processed for immunohistochemistry with Discovery XT from Ventana Medical Systems (Roche) slide staining system. Stained slides were digitized with a NanoZoomer slide scanner (Hamamatsu, Massy, France) and viewed with the NDP.view2 software (Hamamatsu). Signal scoring was independently read by two observers (OM, LC) blinded to any information and discrepancies were resolved by

consensus reading. A five-point scale (0-1-2-3-4) denoting increasing signal intensity was used. Digital images were obtained with either a single focus (Nanozoomer, Hamamatsu Photonics) or a confocal (3D-Histech) slide scanner. To quantify dual ACE2 and ABCC2 staining, indicative of ACE2 expression by biliary pole of tumor hepatocytes in HCCs, we used the HALO Spatial Analysis software (Indica Labs). The design of the quantification algorithm was done by an observer (NM) blinded to any information. Immunohistochemistry (see below), digital imaging and data acquisition were performed in an ISO-9001-certified core facility (High Precision Histopathology, H2P2, CNRS 3840, INSERM 018, Rennes 1 University). Data analysis was performed with R 3.6.2 and the R package dplyr 0.8.5 for data cleaning and transformation. The Mann Whitney U test was used to assess the differences between means.

Immunohistochemistry

The above described TMA and routine formalin-fixed, paraffin-embedded tissue blocks from the Anatomic Pathology laboratory, Rennes University Hospital, were used to prepare 5-µm sections mounted on Super Frost Plus slides (VWR International). Antibodies used, incubation conditions, horseradish peroxidase and tyramide-based signal amplification are summarized in Supplementary Table 6. Negative controls were performed with isotypic immunoglobulins obtained from the same species as the antibodies shown in Supplementary Table 6.

Statistical analysis

Survival analyses were performed using the log rank test and Kaplan Meier Curves (*Survival* R package). Correlations between genes were calculated using either the

Pearson's or Spearman's R methods. Continuous variables were compared by parametric testing (Student's *t*-test and ANOVA) or non-parametric testing (Mann-Whitney U test); whereas categorical data were compared with Fisher's exact test. *P* values were adjusted for multiple testing using the Benjamini-Hochberg procedure.

Weighted Gene Correlation Network Analysis (WGCNA) was performed using the *WGCNA*[2] R package. The network file was then exported to Cytoscape[3]. Functional analysis and GO enrichment were conducted after gene module detection and results were summarized using Express Analysis from Metascape[4] or REVIGO[5], as indicated.

Supplementary Figure legends

Supplementary Fig. 1 **(a)** Exclusion criteria for overall and disease-free survival analyses applied to 370 HCC-resected patients from the TCGA dataset. Numbers of patients excluded in each category are indicated. **(b)** Lower *ACE2* mRNA expression is associated with worse overall and disease-free survival after resection of HCCs (Kaplan-Meier curves with 95% confidence intervals; log-rank test). Patients at risk are indicated for each time point.

Supplementary Fig. 2 **(a)** Lower *TMPRSS2* mRNA expression is associated with worse overall and disease-free survival after resection of HCCs (Kaplan-Meier curves, log-rank test). **(b)** *ACE2* and *TMPRSS2* mRNA expression in 370 HCCs and 47 matching non-tumor livers, extracted from The Cancer Genome Atlas (TCGA) database. Raw RNA sequencing data were extracted in April 2020 and normalized by the median *(DESeq* R package). Box plots show median plus 25 – 75 interquartile range and outliers. The statistical significance of the differences between means was

assessed with the Student's *t* test. **(c)** *ACE2* and *TMPRSS2* mRNA expression in 370 HCCs according to HCC subclasses:[6] PP, periportal-type; PV, perivenous-type; ECM, extracellular-matrix-type and STEM, stem-cell-type HCCs or activating β-catenin *(CTNNB1)* mutations. **(d)** *TMPRSS2* and **(e):** DPP4 mRNA expression in HCC subclasses[6]. **(f)** *DPP4* mRNA expression in HCCs according to the activation of β-catenin transcriptional activity. β-catenin activation was assessed as described[6]. **(g)** *DPP4* mRNA expression in HCCs according to activating *CTNNB1* mutations.

Supplementary Fig. 3 **(a)** *TMPRSS2* DNA methylation levels in HCCs according to *CTNNB1* mutational status in the TCGA dataset. See Supplementary Table 2. **(b)** *TERT* and *TP53* mutation rates in 370 HCCs from the TCGA dataset expressing *high* or *low ACE2, TMPRSS2* and *DPP4* mRNA levels. *High* or *low* mRNA levels in HCCs are those above or below the median. *P* values were calculated with X^2 test. **(c, d):** *ACE2, TMPRSS2* and *DPP4* mRNA levels in 370 HCCs from the TCGA dataset according to *TERT* ***(c)*** and *TP53* ***(d)*** gene mutations. *P* values were calculated with the Student's *t* test with Welch correction. HCCs with mutated *TP53* have lower *TMPRSS2* mRNA levels.

Supplementary Fig. 4 **(a)** TCF/LEF response sequences in human *ACE2*, 5000 bases upstream the transcription start site. **(b)** TCF/LEF response sequences in human *DPP4,* 5000 bases upstream the transcription start site. Putative TCF/LEF-1 binding sites responding to β-catenin transcriptional activation in the proximal DNA sequences were predicted with the PROMO program using the TRANSFAC database[7].

Supplementary Fig. 5 **(a)** Immunoperoxidase detection of TMPRSS2 in HCCs in slit-like structures ***(green arrows)*** and in the lumen of pseudo-glandular formations ***(red arrows)*** and **(b)** in cell-cell borders ***(red arrows)***. Slides were lightly counterstained with hematoxylin ***(blue)****.* **(c)** Detection of ACE2 and ACTA2 (a.k.a. alpha smooth muscle actin) in HCC. ***Left,*** immunoperoxidase ***(brown)*** with light hematoxylin counterstaining ***(blue)****.* ***Middle and right****,* ACE2 and ACTA2 co-immunodetection does not show evidence of ACE2 expression in myofibroblasts within the tumor. Representative images from a tissue microarray containing 41 HCCs and two normal liver controls, spotted in triplicate 1-mm diameter punches. Digital images were acquired with a microscope scanner and at X40 (Nanozoomer, Hamamatsu Photonics).

Supplementary Fig. 6 Positive controls for ACE2 **(a, b)** and TMPRSS2 **(c, d)** antibodies. Immunoperoxidase staining ***(brown)*** and light hematoxylin counterstaining ***(blue)****,* in intestine **(a)**, kidney **(b)** and prostate **(c, d)**. ACE2 is detected in the apical pole of the intestinal epithelium **(a)**. In the kidney, ACE2 positive signal is seen in the parietal cell layer of the Bowman's capsule and the convoluted tubules **(b)**. TMPRSS2 is detected in the apical pole of the glandular epithelium and in surrounding smooth muscle **(c, d)**. Digital images were acquired with a slide scanner and a X20 objective (Nanozoomer, Hamamatsu Photonics) and exported at the following magnifications: **a** and **c,** X5; **b** and **d**, X20.

Supplementary Figure 7 **(a)** The *ACE2* mRNA correlation network in 226 human HCCs with high levels of β-catenin pathway activation applying Pearson's correlation

coefficients (> +0.25 and < -0.25) using the Désert's dataset[6]. The gene network was visualized with Cytoscape 3.7.1, where red-colored ellipses correspond to positive and green-colored ones to negative correlations. The thickness of blue edges is proportional to the absolute value of correlation coefficients. See Supplementary Table 3. **(b)** Interactive network of gene functions associated with *ACE2* mRNA expression in 226 human HCCs with β-catenin pathway activation. It includes carbohydrate and lipid metabolism, cell cycle and cytokine-mediated signaling. Network visualization was built with Revigo[5] using gene ontology terms from Supplementary Tables 4 and 5.

Supplementary Table Legends

Supplementary Table 1 DNA methylation in 291 HCCs carrying wild-type β-catenin *(CTNNB1)* and 79 HCCs carrying mutated *CTNNB1* from the TCGA dataset. Raw RNA sequencing and DNA methylation data were downloaded in April 2020 and median-normalized using the *DESeq* R package. Columns "logFC" (log Fold Change) and "abs" (absolute) describe how differentially methylated probes are between HCCs carrying mutated *CTNNB1* and HCCs carrying wild type *CTNNB1*. A negative log fold-change reflects a probe less methylated in *CTNNB1*-mutated HCCs than in *CTNNB1*-wild type HCCs. "AveExpr" is the average level of methylation for a probe of interest across all the data. *"t"* represents the t-statistic used to assess differential methylation and the B-statistic is the log-odds that a probe is differentially methylated. Column *"P.Value"* contains the unadjusted *p*-values for differential analysis. "*Adj.P.val"* contains *p*-values adjusted for multiple testing using the Benjamini-Hochberg procedure.

Supplementary Table 2 Gene mRNA transcripts correlating with *ACE2* mRNA in 226 human HCCs with high levels of β-catenin pathway activation from Désert's

dataset[6] were obtained with the *Corrplot* R package for Pearson's correlation. *ACE2* co-expression network included liver-enriched genes involved in hepatocyte-specific functions, such as aminoacid metabolism *(GNMT),* xenobiotic detoxification *(CYP3A4),* fatty acid uptake, bile acid reconjugation *(SLC27A5),* enterohepatic circulation of bile acids and cholesterol homeostasis *(SLC10A1),* which is consistent with our finding of ACE2 in bile canaliculi*. ACE2* was also co-expressed with *SIRT1* and *CD36* and the fatty acid oxidation enzymes *ACADL, EHHADH, ACADM and ECI2* and alternative glucose metabolism pathways *(CRYL1),* as well as enzymes involved in gluconeogenesis *(PCK2).* Also, high *ACE2* was associated with low levels of the inflammation-related transcripts *BIRC3, MSN, MMP9, LTB, TNFRSF4, IL4R, CCL20, IL32,* among others.

Supplementary Table 3 Ontology of genes correlating with *ACE2* (correlation coefficient > +0.25) in 226 human HCCs with high levels of β-catenin pathway activation from Désert's dataset[6], using FuncAssociate 3.0 web-based software[8].

Supplementary Table 4 Ontology of genes correlating with *ACE2* (correlation coefficient < -0.25) in 226 human HCCs with high levels of β-catenin pathway activation from Désert's dataset[6], using FuncAssociate 3.0 web-based software[8].

Supplementary Table 5 Activating mutations in the 3rd exon of the *CTNNB1* gene (β-catenin phosphorylation hotspot)[9]; *1,* mutated, *0,* wild-type, as assessed by Sanger sequencing[10]; mean GLUL and ACE2 protein expression scores as assessed by immunohistochemistry in a tissue microarray (TMA) composed of 41 HCCs and two normal liver controls, as shown in Figure 1E-H. *SPOT* denotes TMA location of triplicate 1 mm in diameter formalin-fixed-paraffin-embedded liver tissue cores, beginning from the lower right corner in Figure 1E and F. Rows, A to I, from bottom to top; columns, 1 to 15, from right to left. Please note that rows A and B contain 12 spots,

rows C to I contain 15 spots. β-catenin activation score was calculated using the following formula:

β-catenin activation score = *(GLUL x LGR5 x ODAM) ÷ (VNN1 x HAL),*

where the mRNA expression of the indicated genes was analyzed by real-time PCR by the $2^{-\Delta\Delta Ct}$ method. The mRNA expression dataset for the indicated genes, samples, primer sequences and PCR methods were previously published by our group showing that the score predicted β-catenin activation with high sensitivity and specificity[6] (AUROC 0.87 to 0.92; sensitivity, 0.86 to 0.91; specificity, 0.83 to 1 in training and validation sets)[6]. Here, β-catenin activation scores above 4 (median) were defined as *high*, thereby denoting the perivenous phenotype in HCCs, according to our previously published data[6,11].

Supplementary Table 6 Antibodies and immunohistochemical methods. For a full description of immunohistochemical methods see *Immunohistochemistry* in the *Supplementary Methods* section above.

REFERENCES

1 Desert, R. *et al.* "Fibrous nests" in human hepatocellular carcinoma express a Wnt-induced gene signature associated with poor clinical outcome. *Int J Biochem Cell Biol* **81(Pt A)**, 195-207, doi:10.1016/j.biocel.2016.08.017 (2016).

2 Langfelder, P. & Horvath, S. WGCNA: an R package for weighted correlation network analysis. *BMC Bioinformatics* **9**, 559, doi:10.1186/1471-2105-9-559 (2008).

3 Shannon, P. *et al.* Cytoscape: a software environment for integrated models of biomolecular interaction networks. *Genome Res* **13**, 2498-2504, doi:10.1101/gr.1239303 (2003).

4 Zhou, Y. *et al.* Metascape provides a biologist-oriented resource for the analysis of systems-level datasets. *Nat Commun* **10**, 1523, doi:10.1038/s41467-019-09234-6 (2019).

5 Supek, F., Bosnjak, M., Skunca, N. & Smuc, T. REVIGO summarizes and visualizes long lists of gene ontology terms. *PloS one* **6**, e21800, doi:10.1371/journal.pone.0021800 (2011).

6 Desert, R. *et al.* Human hepatocellular carcinomas with a periportal phenotype have the lowest potential for early recurrence after curative resection. *Hepatology* **66**, 1502-1518, doi:10.1002/hep.29254 (2017).

7 Farre, D. *et al.* Identification of patterns in biological sequences at the ALGGEN server: PROMO and MALGEN. *Nucleic Acids Res* **31**, 3651-3653 (2003).

8 Berriz, G. F., Beaver, J. E., Cenik, C., Tasan, M. & Roth, F. P. Next generation software for functional trend analysis. *Bioinformatics* **25**, 3043-3044, doi:10.1093/bioinformatics/btp498 (2009).

9 de La Coste, A. *et al.* Somatic mutations of the beta-catenin gene are frequent in mouse and human hepatocellular carcinomas. *Proc Natl Acad Sci U S A* **95**, 8847-8851 (1998).

10 Mebarki, S. *et al.* De novo HAPLN1 expression hallmarks Wnt-induced stem cell and fibrogenic networks leading to aggressive human hepatocellular carcinomas. *Oncotarget* **7**, 39026-39043, doi:10.18632/oncotarget.9346 (2016).

11 Desert, R., Nieto, N. & Musso, O. Dimensions of hepatocellular carcinoma phenotypic diversity. *World journal of gastroenterology* **24**, 4536-4547, doi:10.3748/wjg.v24.i40.4536 (2018).
