# Supplementary material for: Well-differentiated liver cancers reveal the potential link between ACE2 dysfunction and metabolic breakdown
Source: Sci Rep. 2022 Feb 3;12:1859. doi: 10.1038/s41598-021-03710-0 (PMC8814043; doi:10.1038/s41598-021-03710-0)
Supplement: Supplementary file 2 — Supplementary Figure 1. [file 41598_2021_3710_MOESM2_ESM.pdf]

**a**

**Exclusion criteria for survival analysis applied to HCC-resected patients from the TCGA dataset**

Whole dataset
370 HCC patients

↓

No total survival, n= 3
No disease-free survival, n= 60
Distant metastasis, n= 3
Lymph node metastasis, n= 3
Previous cancer, n= 27
Resection margin R1, n= 13
Liver transplantation, n= 1
Early death (< 30 days after resection), n= 1
Alive, but no endpoint date available, n= 3

↓

Survival dataset
256 HCC patients

**b**

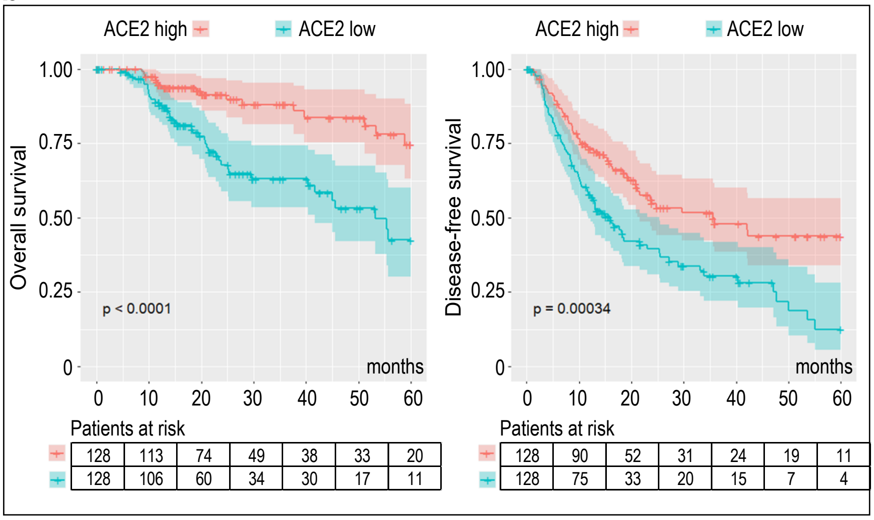


Desquilles et al., Supplementary Fig. 1
