# Supplementary material for: Well-differentiated liver cancers reveal the potential link between ACE2 dysfunction and metabolic breakdown
Source: Sci Rep. 2022 Feb 3;12:1859. doi: 10.1038/s41598-021-03710-0 (PMC8814043; doi:10.1038/s41598-021-03710-0)
Supplement: Supplementary file 3 — Supplementary Figure 2. [file 41598_2021_3710_MOESM3_ESM.pdf]

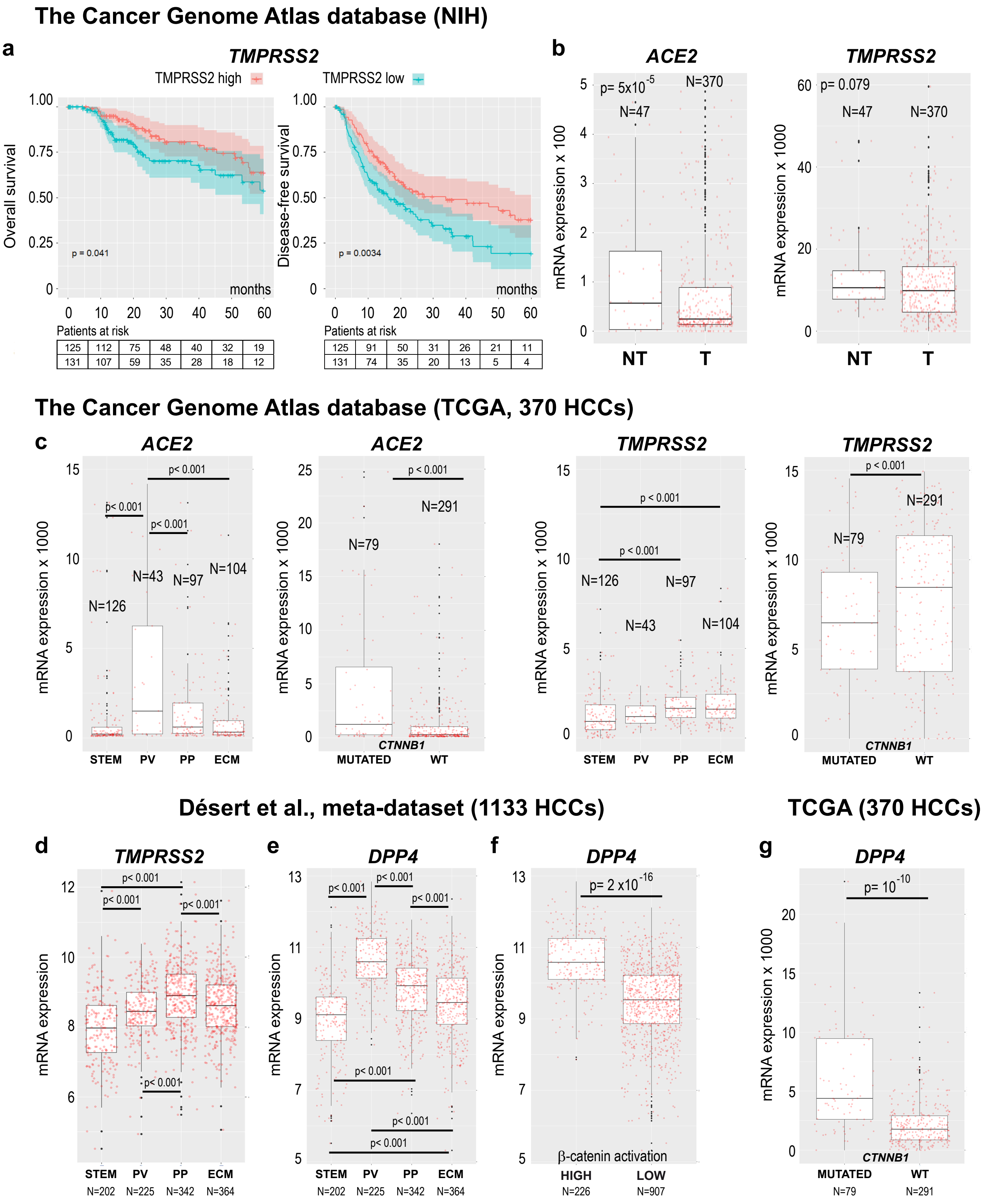

Desquilles et al., Supplementary Fig. 2
