# Supplementary figures and images for: Well-differentiated liver cancers reveal the potential link between ACE2 dysfunction and metabolic breakdown
Source: Sci Rep. 2022 Feb 3;12:1859. doi: 10.1038/s41598-021-03710-0 (PMC8814043; doi:10.1038/s41598-021-03710-0)

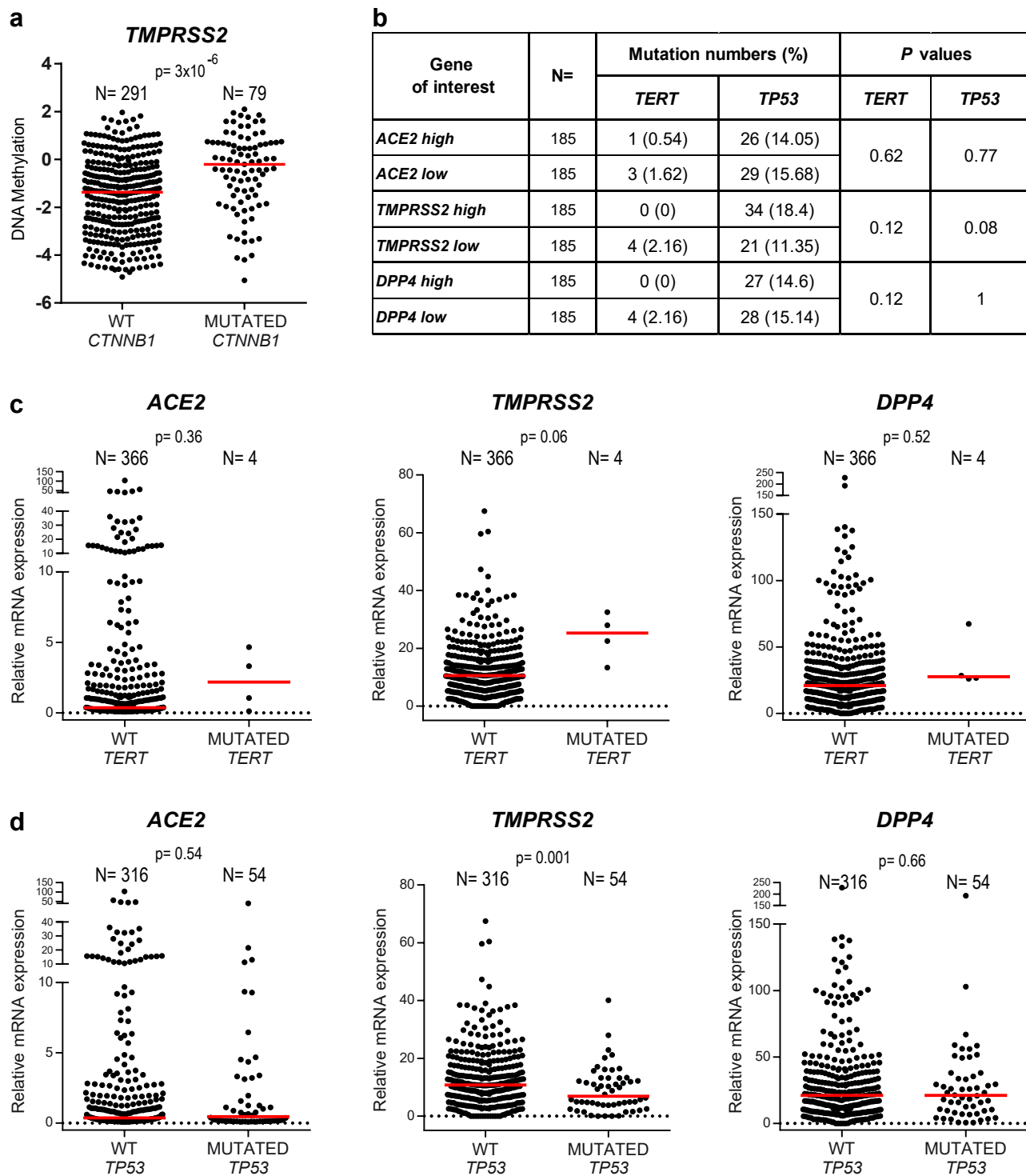

Supplement: Supplementary file 4 — Supplementary Figure 3. [file 41598_2021_3710_MOESM4_ESM.pdf]

a

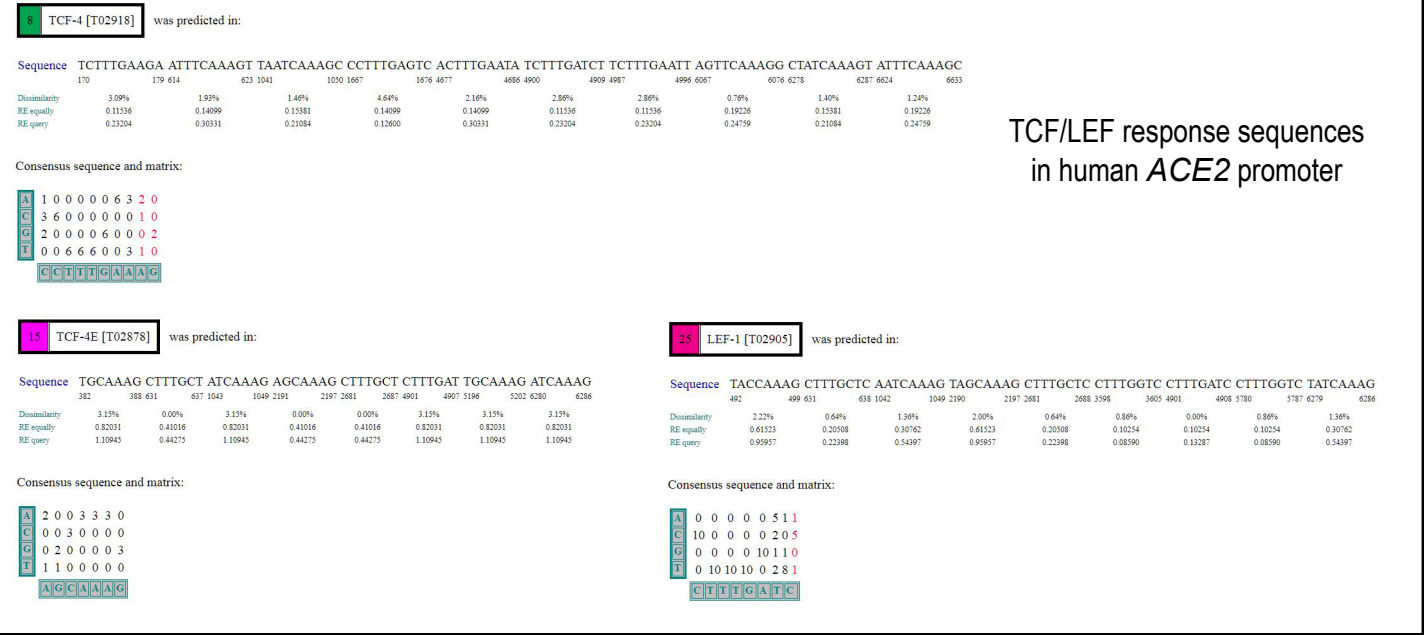

TCF/LEF response sequences  
in human *ACE2* promoter

b

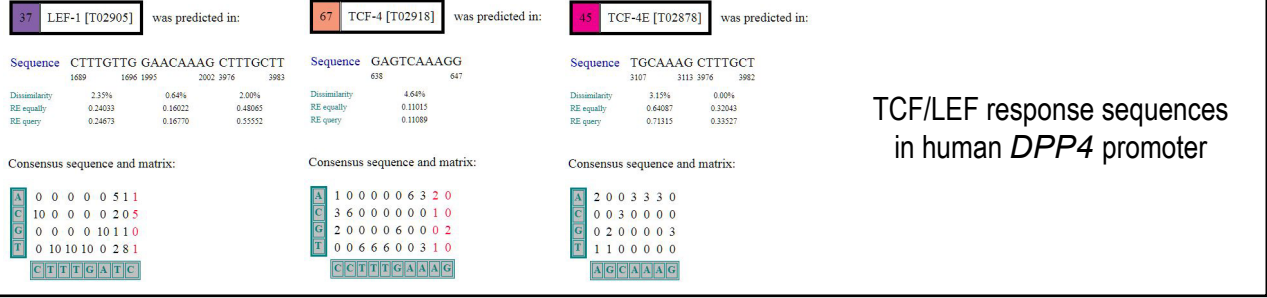

Supplement: Supplementary file 5 — Supplementary Figure 4. [file 41598_2021_3710_MOESM5_ESM.pdf]

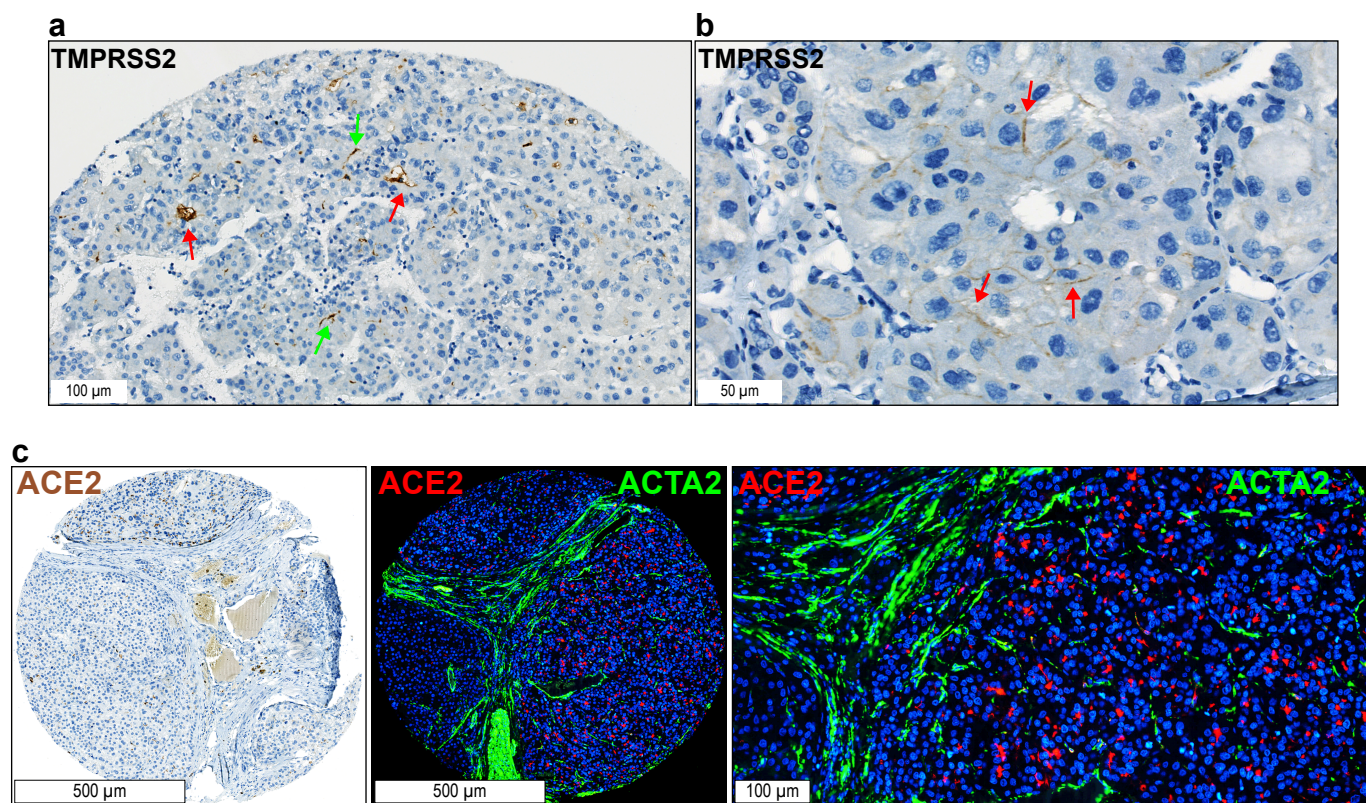

Desquilles et al., Supplementary Fig. 5

Supplement: Supplementary file 6 — Supplementary Figure 5. [file 41598_2021_3710_MOESM6_ESM.pdf]

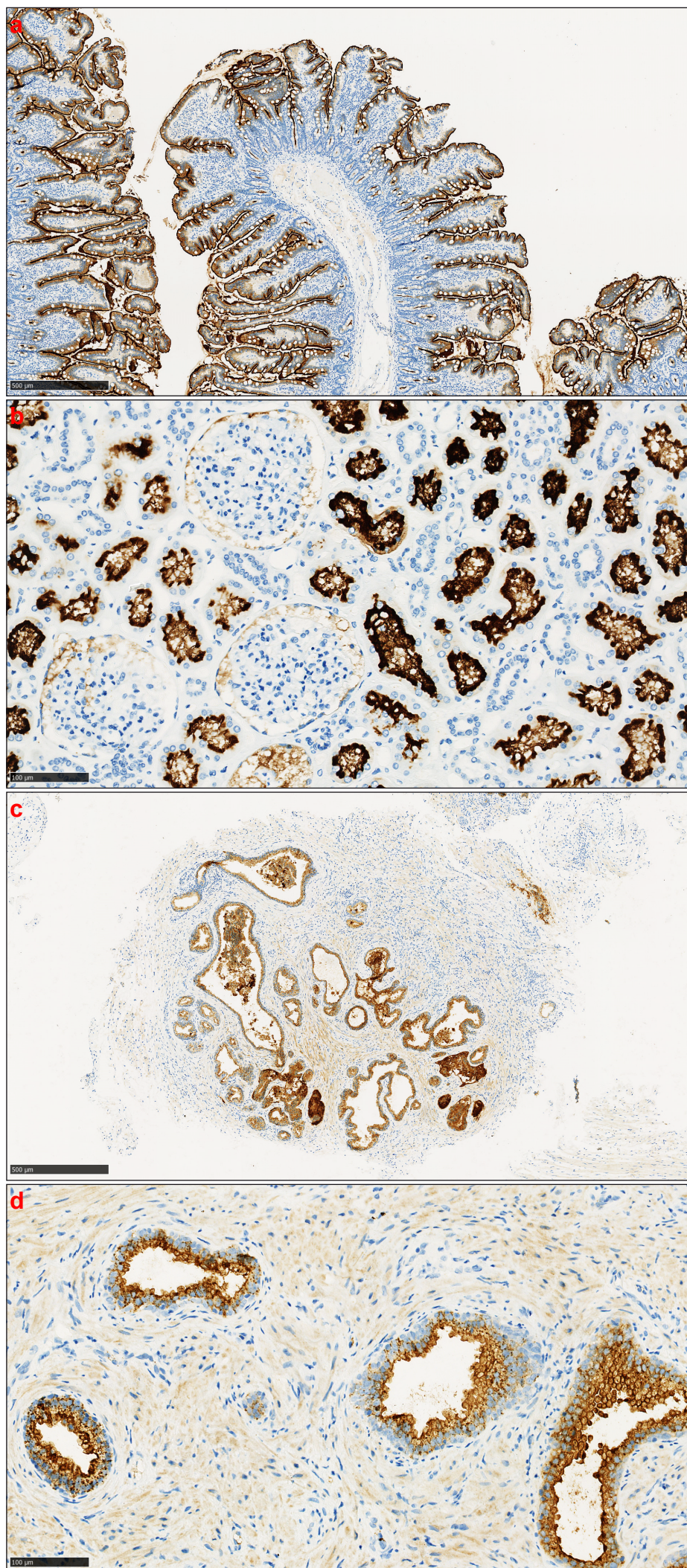

Desquilles et al., Supplementary Fig. 6

Supplement: Supplementary file 7 — Supplementary Figure 6. [file 41598_2021_3710_MOESM7_ESM.pdf]

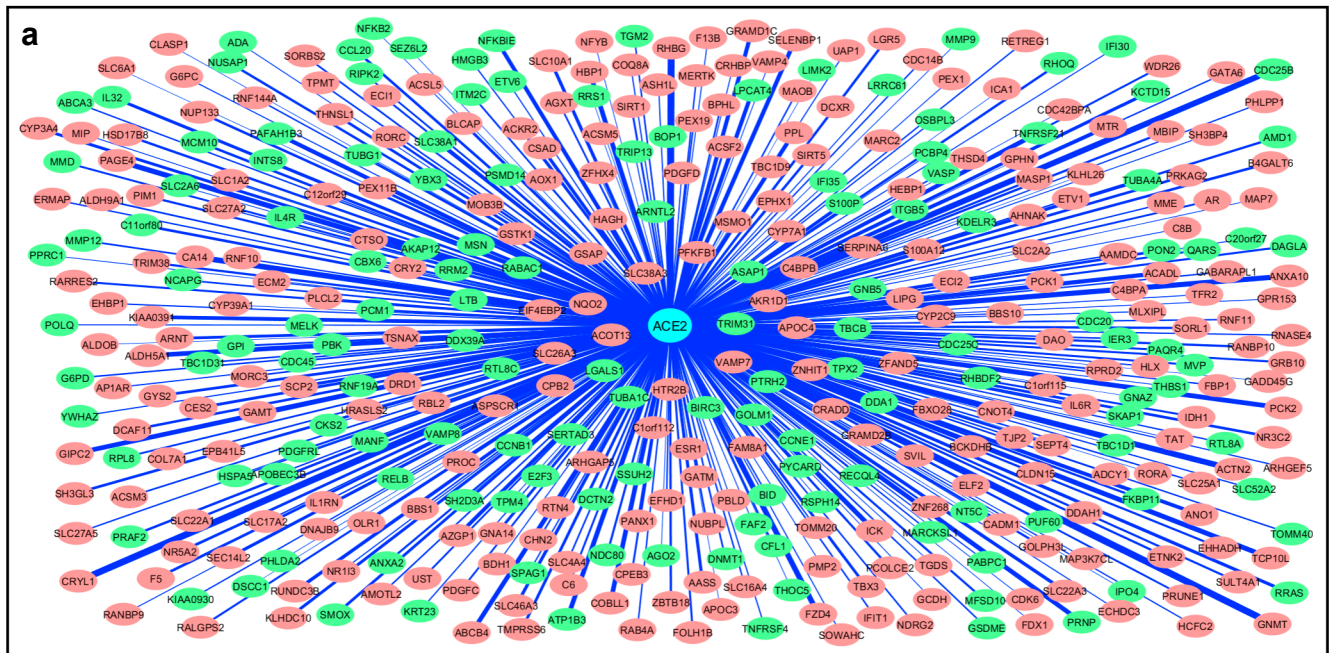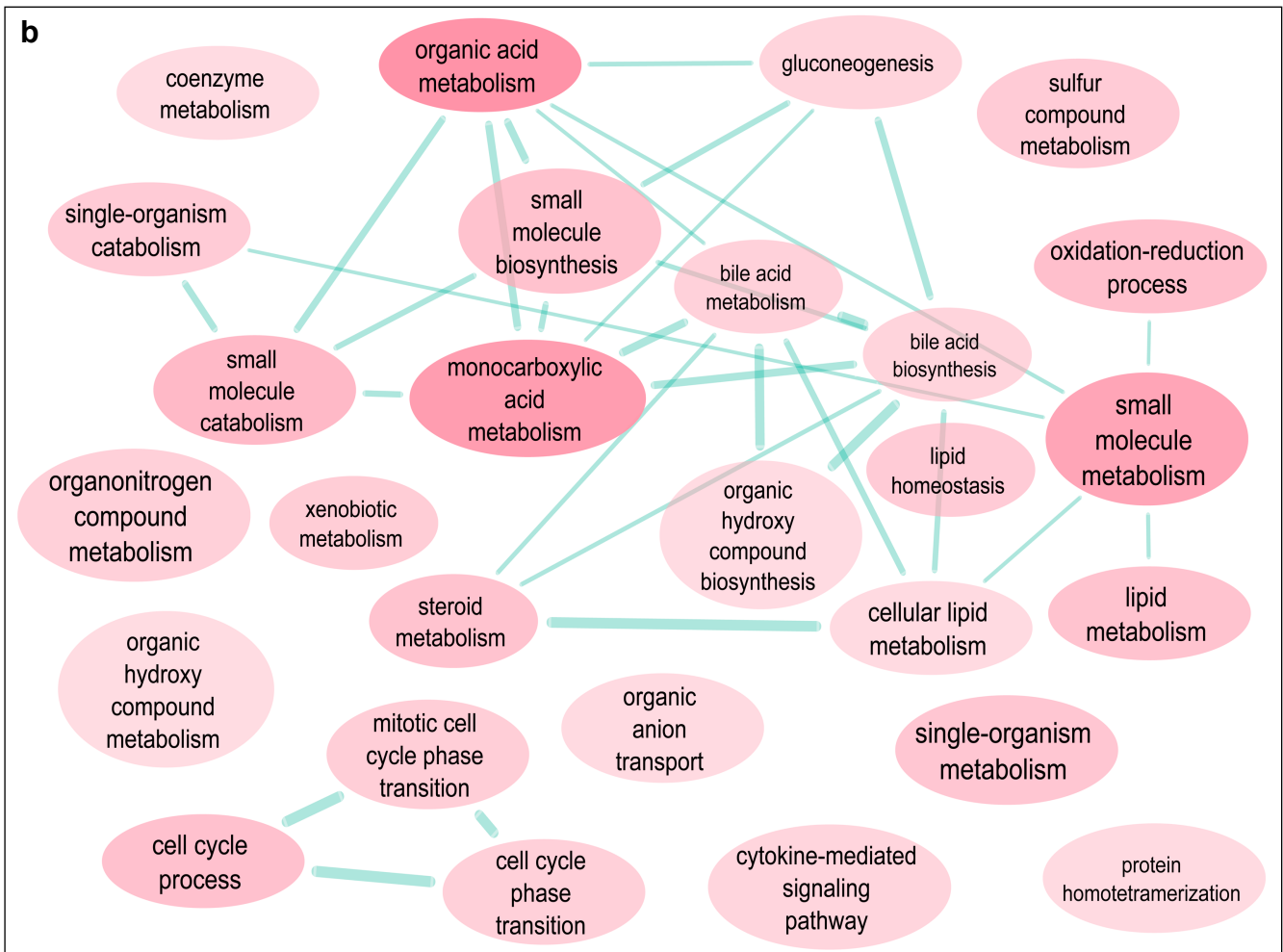

Supplement: Supplementary file 8 — Supplementary Figure 7. [file 41598_2021_3710_MOESM8_ESM.pdf]
