# Supplementary material for: Well-differentiated liver cancers reveal the potential link between ACE2 dysfunction and metabolic breakdown
Source: Sci Rep. 2022 Feb 3;12:1859. doi: 10.1038/s41598-021-03710-0 (PMC8814043; doi:10.1038/s41598-021-03710-0)
Supplement: Supplementary file 9 — Supplementary Table 1. [file 41598_2021_3710_MOESM9_ESM.pdf]

| DNA methylation in 291 HCCs carrying wild-type CTNNB1 and 79 HCCs carrying mutated *CTNNB1* from the TCGA dataset | | | | | | | |
|---|---|---|---|---|---|---|---|
| **Gene symbol** | **logFC** | **AveExpr** | ***t*** | **P.Value** | **adj.P.Val** | **B** | **abs** |
| ***TMPRSS2*** | 1,030306228 | -1,302330529 | 5,423771614 | 9,78E-08 | 3,27E-06 | 5,922974484 | 1,030306228 |
| ***ACE2*** | -0,602063465 | 0,636097685 | -4,558113165 | 6,74E-06 | 9,21E-05 | 1,834523098 | 0,602063465 |
| ***DPP4*** | 0,472521117 | 2,05063653 | 4,344378674 | 1,75E-05 | 0,000195669 | 0,923653403 | 0,472521117 |
