# Supplementary material for: Well-differentiated liver cancers reveal the potential link between ACE2 dysfunction and metabolic breakdown
Source: Sci Rep. 2022 Feb 3;12:1859. doi: 10.1038/s41598-021-03710-0 (PMC8814043; doi:10.1038/s41598-021-03710-0)
Supplement: Supplementary file 11 — Supplementary Table 3. [file 41598_2021_3710_MOESM11_ESM.pdf]

Ontology of genes correlating with ACE2 (correlation coefficient > +0.25) in 226 human hepatocellular carcinomas expressing β-catenin activation signatures (Désert's dataset of 1133 human HCCs)

species: Homo sapiens
support: EXP, IMP, ISS, ISM, TAS, IEA, HMP, HEP, IDA, IGI, ISO, IGC, NAS, HGI, IPI, IEP, ISA, RCA, IC, HDA
associations retrieved on: 2020-04-25 04:31:06-04
gofunc built on: 2018-02-21 10:04:34.764444-05
gene associations file: ftp://ftp.geneontology.org/pub/go/gene-associations/goa_human.gaf.gz
revision: 2018-02-02 $
downloaded on: 2018-02-14 09:47:26-05
GO DAG files from: http://archive.geneontology.org/latest-full/go_201512-termdb-tables.tar.gz
downloaded on: 2015-12-22 05:43:21-05
translation required: yes
translation info:
authority: ensembl
synergizer db built on: 2015-11-17 14:24:21.26681-05
synergizer db built from: Ensembl Genes 82 / Metazoa Mart / Plant Mart
namespace: hgnc_symbol
coverage: 99.8%
size of "genespace": 18224
number of attributes: 20270
NOTE: the last record consists of all the entities in the
"genespace" that are not associated with any attribute (for
the requested support).
Original number of entities in query: 253
Number of entities in query: 253
Total number of entities: 18232
Original total number of entities: 18224
Entities not in default gene space: 8
Total number of attributes: 20270
Translation required: yes
Translation coverage: 0.997782
Mode: unordered
Number of simulations: 1000
Adjusted P-value cutoff: 0.05
Over/under: both
Evidence codes: EXP, IMP, ISS, ISM, TAS, IEA, HMP, HEP, IDA, IGI, ISO, IGC, NAS, HGI, IPI, IEP, ISA, RCA, IC, HDA

OVERREPRESENTED ATTRIBUTES

N	X	LOD	P	P_adj	attrib ID	attrib name
3	3	2,70106792	2,64E-06	0,01	GO:0004165	dodecenoyl-CoA delta-isomerase activity
5	20	1,409119732	6,47E-06	0,019	GO:0016878	acid-thiol ligase activity
6	27	1,341169752	1,56E-06	0,001	GO:0006699	bile acid biosynthetic process
7	37	1,252996856	6,58E-07	0,001	GO:0008206	bile acid metabolic process
7	42	1,186947349	1,63E-06	0,001	GO:0006094	gluconeogenesis
7	45	1,15164236	2,64E-06	0,006	GO:0019319	hexose biosynthetic process
6	39	1,14827311	1,50E-05	0,046	GO:0016877	ligase activity, forming carbon-sulfur bonds
7	50	1,098492785	5,48E-06	0,017	GO:0046364	monosaccharide biosynthetic process
14	102	1,087765967	1,38E-10	<0.001	GO:0072329	monocarboxylic acid catabolic process
11	81	1,080880442	1,52E-08	<0.001	GO:0005496	steroid binding
7	52	1,078922217	7,16E-06	0,021	GO:0006635	fatty acid beta-oxidation
7	54	1,06019157	9,26E-06	0,025	GO:0003707	steroid hormone receptor activity
11	96	0,996739551	9,22E-08	<0.001	GO:0055088	lipid homeostasis
11	97	0,991665288	1,03E-07	<0.001	GO:0005777	peroxisome
10	90	0,981735353	4,79E-07	0,001	GO:0044438	microbody part
10	90	0,981735353	4,79E-07	0,001	GO:0044439	peroxisomal part
11	102	0,967138929	1,73E-07	<0.001	GO:0006805	xenobiotic metabolic process
8	76	0,956808815	1,03E-05	0,025	GO:0051289	protein homotetramerization
12	118	0,938852875	9,13E-08	<0.001	GO:0042579	microbody
8	79	0,938120599	1,37E-05	0,045	GO:0009062	fatty acid catabolic process
19	208	0,892470704	9,70E-11	<0.001	GO:0016054	organic acid catabolic process
19	208	0,892470704	9,70E-11	<0.001	GO:0046395	carboxylic acid catabolic process
11	120	0,888708682	9,05E-07	0,001	GO:0006694	steroid biosynthetic process
16	179	0,879124965	4,18E-09	<0.001	GO:0050662	coenzyme binding
19	241	0,821943572	1,19E-09	<0.001	GO:0048037	cofactor binding
18	244	0,789396595	9,43E-09	<0.001	GO:0008202	steroid metabolic process
32	465	0,773711029	7,81E-14	<0.001	GO:0032787	monocarboxylic acid metabolic process
11	156	0,764384042	1,17E-05	0,041	GO:0072330	monocarboxylic acid biosynthetic process
23	337	0,757902217	3,56E-10	<0.001	GO:0044282	small molecule catabolic process
18	274	0,734642937	5,64E-08	<0.001	GO:0006631	fatty acid metabolic process
12	188	0,717753557	1,29E-05	0,042	GO:1901617	organic hydroxy compound biosynthetic process
19	318	0,69098948	1,07E-07	<0.001	GO:0006790	sulfur compound metabolic process
15	252	0,686194515	2,54E-06	0,006	GO:0016042	lipid catabolic process
43	787	0,679796341	1,00E-14	<0.001	GO:0019752	carboxylic acid metabolic process
14	239	0,678224385	6,63E-06	0,019	GO:0016053	organic acid biosynthetic process
14	239	0,678224385	6,63E-06	0,019	GO:0046394	carboxylic acid biosynthetic process
24	426	0,668574616	6,68E-09	<0.001	GO:0044283	small molecule biosynthetic process
47	908	0,659983979	3,66E-15	<0.001	GO:0006082	organic acid metabolic process
46	891	0,657192676	8,31E-15	<0.001	GO:0043436	oxoacid metabolic process
14	256	0,64624315	1,45E-05	0,045	GO:0006732	coenzyme metabolic process
18	338	0,636331186	1,25E-06	0,001	GO:0005759	mitochondrial matrix
18	387	0,573338061	8,30E-06	0,022	GO:0015711	organic anion transport
36	857	0,544705354	2,87E-09	<0.001	GO:0055114	oxidation-reduction process
31	770	0,518592217	1,05E-07	<0.001	GO:0044712	single-organism catabolic process
20	486	0,517988275	1,58E-05	0,046	GO:1901615	organic hydroxy compound metabolic process
61	1660	0,514787292	9,20E-13	<0.001	GO:0044281	small molecule metabolic process
25	653	0,488685719	4,74E-06	0,015	GO:0016491	oxidoreductase activity
42	1159	0,481752989	9,41E-09	<0.001	GO:0006629	lipid metabolic process
31	898	0,446024516	2,80E-06	0,011	GO:0070013	intracellular organelle lumen
30	908	0,424314751	9,70E-06	0,025	GO:0044255	cellular lipid metabolic process
45	1474	0,402532296	4,00E-07	0,001	GO:1901564	organonitrogen compound metabolic process
34	1093	0,399696802	8,56E-06	0,024	GO:0043233	organelle lumen
36	1167	0,397740287	5,44E-06	0,017	GO:0031974	membrane-enclosed lumen
75	2739	0,385824186	1,98E-09	<0.001	GO:1903561	extracellular vesicle
75	2740	0,385632867	2,01E-09	<0.001	GO:0043230	extracellular organelle
74	2721	0,380868824	3,60E-09	<0.001	GO:0070062	extracellular exosome
74	2722	0,38067649	3,66E-09	<0.001	GO:0065010	extracellular membrane-bounded organelle
79	3118	0,350134681	2,17E-08	<0.001	GO:0031988	membrane-bounded vesicle
86	3563	0,333177032	3,72E-08	<0.001	GO:0044421	extracellular region part
80	3303	0,327152089	1,25E-07	<0.001	GO:0031982	vesicle

95	4074	0,326289142	2,63E-08	<0.001	GO:0044710	single-organism metabolic process
101	4931	0,258113352	5,26E-06	0,017	GO:0003824	catalytic activity
160	9049	0,244373863	7,86E-06	0,021	GO:0043227	membrane-bounded organelle

UNDERREPRESENTED ATTRIBUTES

N	X	LOD	P	P_adj	attrib ID	attrib name
