# Supplementary material for: Well-differentiated liver cancers reveal the potential link between ACE2 dysfunction and metabolic breakdown
Source: Sci Rep. 2022 Feb 3;12:1859. doi: 10.1038/s41598-021-03710-0 (PMC8814043; doi:10.1038/s41598-021-03710-0)
Supplement: Supplementary file 12 — Supplementary Table 4. [file 41598_2021_3710_MOESM12_ESM.pdf]

Ontology of genes correlating with ACE2 (correlation coefficient < -0.25) in 226 human hepatocellular carcinomas expressing β-catenin activation signatures (Désert's dataset of 1133 human HCCs)

species: Homo sapiens
support: EXP, IMP, ISS, ISM, TAS, IEA, HMP, HEP, IDA, IGI, ISO, IGC, NAS, HGI, IPI, IEP, ISA, RCA, IC, HDA
associations retrieved on: 2020-04-25 04:48:24-04
gofunc built on: 2018-02-21 10:04:34.764444-05
gene associations file: ftp://ftp.geneontology.org/pub/go/gene-associations/goa_human.gaf.gz
revision: 2018-02-02 $
downloaded on: 2018-02-14 09:47:26-05
GO DAG files from: http://archive.geneontology.org/latest-full/go_201512-termdb-tables.tar.gz
downloaded on: 2015-12-22 05:43:21-05
translation required: yes
translation info:
authority: ensembl
synergizer db built on: 2015-11-17 14:24:21.26681-05
synergizer db built from: Ensembl Genes 82 / Metazoa Mart / Plant Mart
namespace: hgnc_symbol
coverage: 99.8%
size of "genespace": 18224
number of attributes: 20270
NOTE: the last record consists of all the entities in the
"genespace" that are not associated with any attribute (for
the requested support).
Original number of entities in query: 141
Number of entities in query: 141
Total number of entities: 18230
Original total number of entities: 18224
Entities not in default gene space: 6
Total number of attributes: 20270
Translation required: yes
Translation coverage: 0.997782
Mode: unordered
Number of simulations: 1000
Adjusted P-value cutoff: 0.05
Over/under: both
Evidence codes: EXP, IMP, ISS, ISM, TAS, IEA, HMP, HEP, IDA, IGI, ISO, IGC, NAS, HGI, IPI, IEP, ISA, RCA, IC, HDA

OVERREPRESENTED ATTRIBUTES

**N**	**X**	**LOD**	**P**	**P_adj**	**attrib ID**	**attrib name**
8	129	0,973873206	7,28E-06	0,022	GO:0000086	G2/M transition of mitotic cell cycle
8	131	0,966734184	8,15E-06	0,022	GO:0044839	cell cycle G2/M phase transition
12	233	0,891354611	2,67E-07	0,001	GO:0044772	mitotic cell cycle phase transition
12	241	0,875751167	3,84E-07	0,002	GO:0044770	cell cycle phase transition
24	601	0,800924377	3,59E-11	<0.001	GO:1903047	mitotic cell cycle process
27	966	0,641907209	5,19E-09	0,001	GO:0022402	cell cycle process
18	638	0,62504346	2,15E-06	0,015	GO:0019221	cytokine-mediated signaling pathway
66	4767	0,399370176	9,73E-08	0,001	GO:0005829	cytosol
38	2445	0,38436938	1,42E-05	0,034	GO:0043228	non-membrane-bounded organelle
38	2445	0,38436938	1,42E-05	0,034	GO:0043232	intracellular non-membrane-bounded organelle
108	10883	0,342132144	1,65E-05	0,043	GO:0005515	protein binding

UNDERREPRESENTED ATTRIBUTES

N	X	LOD	P	P_adj	attrib ID	attrib name
