# Supplementary material for: Well-differentiated liver cancers reveal the potential link between ACE2 dysfunction and metabolic breakdown
Source: Sci Rep. 2022 Feb 3;12:1859. doi: 10.1038/s41598-021-03710-0 (PMC8814043; doi:10.1038/s41598-021-03710-0)
Supplement: Supplementary file 13 — Supplementary Table 5. [file 41598_2021_3710_MOESM13_ESM.pdf]

SPOT	SAMPLE TYPE	CTNNB1 MUTATION EXON 3	GLUL MEAN SCORES	ACE2 MEAN SCORES	β-catenin ACTIVATION SCORE
A01<br>A02<br>A03	NORMAL LIVER CONTROL	not applied	unavailable spot<br>not applied	unavailable spot<br>not applied<br>unavailable spot	0
A04<br>A05<br>A06	NORMAL LIVER CONTROL	not applied	not applied	unavailable spots	0
A07<br>A08<br>A09	HCC	1	0.0	2.0	1
A10<br>A11<br>A12	HCC	0	0.0	1.0	9
B01<br>B02<br>B03	HCC	1	0.0	0.0	0
B04<br>B05<br>B06	HCC	1	2.5	0.0	9
B07<br>B08<br>B09	HCC	1	2.0	1.0	0
B10<br>B11<br>B12	HCC	0	1.0	1.0	6
C01<br>C02<br>C03	HCC	1	0.0	3.0	9
C04<br>C05<br>C06	HCC	1	2.0	2.0	12
C07<br>C08<br>C09	HCC	1	3.0	3.0	8
C10<br>C11<br>C12	HCC	1	4.0	3.0	9
C13<br>C14<br>C15	HCC	0	0.0	1.0	6
D01<br>D02<br>D03	HCC	1	1.7	1.0	3
D04<br>D05<br>D06	HCC	1	3.0	unavailable spots	12
D07<br>D08<br>D09	HCC	1	4.0	4.0	16
D10<br>D11<br>D12	HCC	1	3.0	2.0	4
D13<br>D14<br>D15	HCC	1	3.0	3.0	16
E01<br>E02<br>E03	HCC	1	3.0	3.0	6
E04<br>E05<br>E06	HCC	1	2.5	3.0	12
E07<br>E08<br>E09	HCC	1	4.0	4.0	20
E10<br>E11<br>E12	HCC	1	2.5	3.0	16
E13<br>E14<br>E15	HCC	1	4.0	3.0	16
F01<br>F02<br>F03	HCC	0	2.5	0.0	16
F04<br>F05<br>F06	HCC	0	0.0	0.0	0
F07<br>F08<br>F09	HCC	0	1.0	1.0	3
F10<br>F11<br>F12	HCC	0	0.0	0.0	0
F13<br>F14<br>F15	HCC	0	0.0	1.0	-2
G01<br>G02<br>G03	HCC	0	0.0	unavailable spots	0
G04<br>G05<br>G06	HCC	0	0.0	0.0	0
G07<br>G08<br>G09	HCC	0	1.5	1.2	9
G10<br>G11<br>G12	HCC	0	0.0	0.5	2
G13<br>G14<br>G15	HCC	0	0.0	0.0	4
H01<br>H02<br>H03	HCC	0	0.3	0.3	6
H04<br>H05<br>H06	HCC	0	1.0	1.7	-1
H07<br>H08<br>H09	HCC	1	4.0	1.0	16
H10<br>H11<br>H12	HCC	0	1.7	1.7	-2
H13<br>H14<br>H15	HCC	0	0.0	unavailable spots	0
I01<br>I02<br>I03	HCC	0	0.0	0.0	0
I04<br>I05<br>I06	HCC	0	1.0	0.0	0
I07<br>I08<br>I09	HCC	0	0.0	1.0	-2
I10<br>I11<br>I12	HCC	0	0.0	1.0	-1
I13<br>I14<br>I15	HCC	0	1.0	1.3	4
