# Supplementary material for: Well-differentiated liver cancers reveal the potential link between ACE2 dysfunction and metabolic breakdown
Source: Sci Rep. 2022 Feb 3;12:1859. doi: 10.1038/s41598-021-03710-0 (PMC8814043; doi:10.1038/s41598-021-03710-0)
Supplement: Supplementary file 14 — Supplementary Table 6. [file 41598_2021_3710_MOESM14_ESM.pdf]

Specificity	Clone	Isotype	Antigen retrieval	Stainer kit	Signal amplification	Incubation conditions	Supplier	Dilution	Secondary Antibody	Test type
**ACE2**	CL4035	IgG1	pH8 - Ultra Cell Conditional Solution (Ultra CC1)	Ventana Omnimap	Non used	60 min, 37°C	Invitrogen	1/800	anti-mouse HRP	immunoperoxidase
**TMPRSS2**	P5H9-A3	IgG1-Kappa	pH8 - Ultra Cell Conditional Solution (Ultra CC1)	Ventana Omnimap	Non used	60 min, 37°C	Merck	1/200	anti-mouse HRP	immunoperoxidase
**ACE2**	CL4035	IgG	pH8 - Ultra Cell Conditional Solution (Ultra CC1)	Ventana Rhodamine kit	HRP enzyme kit Tyramide signal amplification	60 min, 37°C	Invitrogen	1/800	anti-mouse HRP	immunofluorescence
**CD34**	SI16-01	IgG	pH8 - Ultra Cell Conditional Solution (Ultra CC1)	Ventana Fam kit	HRP enzyme kit Tyramide signal amplification	60 min, 37°C	Invitrogen	1/500	anti-rabit-HRP	immunofluorescence
**CLEC4M (DC-SIGNR)**	EPR11211	IgG	pH8 - Ultra Cell Conditional Solution (Ultra CC1)	Ventana Fam kit	HRP enzyme kit Tyramide signal amplification	60 min, 37°C	Abcam	1/750	anti-rabit-HRP	immunofluorescence
**ABCC2 (MRP2)**	EPR10997(2)	IgG	pH8 - Ultra Cell Conditional Solution (Ultra CC1)	Ventana Fam kit	HRP enzyme kit Tyramide signal amplification	60 min, 37°C	Abcam	1/500	anti-rabit-HRP	immunofluorescence
**ACTA2 (α-SMA)**	1A4	IgG2a Kappa	pH8 - Ultra Cell Conditional Solution (Ultra CC1)	Ventana Fam kit	HRP enzyme kit Tyramide signal amplification	60 min, 37°C	Invitrogen	1/200	anti-mouse HRP	immunofluorescence
